# Towards evolutionary predictions: Current promises and challenges

**DOI:** 10.1111/eva.13513

**Published:** 2022-12-09

**Authors:** Meike T. Wortel, Deepa Agashe, Susan F. Bailey, Claudia Bank, Karen Bisschop, Thomas Blankers, Johannes Cairns, Enrico Sandro Colizzi, Davide Cusseddu, Michael M. Desai, Bram van Dijk, Martijn Egas, Jacintha Ellers, Astrid T. Groot, David G. Heckel, Marcelle L. Johnson, Ken Kraaijeveld, Joachim Krug, Liedewij Laan, Michael Lässig, Peter A. Lind, Jeroen Meijer, Luke M. Noble, Samir Okasha, Paul B. Rainey, Daniel E. Rozen, Shraddha Shitut, Sander J. Tans, Olivier Tenaillon, Henrique Teotónio, J. Arjan G. M. de Visser, Marcel E. Visser, Renske M. A. Vroomans, Gijsbert D. A. Werner, Bregje Wertheim, Pleuni S. Pennings

**Affiliations:** ^1^ Swammerdam Institute for Life Sciences University of Amsterdam Amsterdam The Netherlands; ^2^ National Centre for Biological Sciences Bangalore India; ^3^ Clarkson University Potsdam New York USA; ^4^ Institute of Ecology and Evolution University of Bern Bern Switzerland; ^5^ Swiss Institute of Bioinformatics Lausanne Switzerland; ^6^ Gulbenkian Science Institute Oeiras Portugal; ^7^ Institute for Biodiversity and Ecosystem Dynamics University of Amsterdam Amsterdam The Netherlands; ^8^ Origins Center Groningen The Netherlands; ^9^ Laboratory of Aquatic Biology, KU Leuven Kulak Kortrijk Belgium; ^10^ University of Helsinki Helsinki Finland; ^11^ Mathematical Institute Leiden University Leiden The Netherlands; ^12^ Instituto Gulbenkian de Ciência Oeiras Portugal; ^13^ Harvard University Cambridge Massachusetts USA; ^14^ Max Planck Institute for Evolutionary Biology Plön Germany; ^15^ Department of Ecological Science Vrije Universiteit Amsterdam Amsterdam The Netherlands; ^16^ Max Planck Institute for Chemical Ecology Jena Germany; ^17^ Netherlands Institute of Ecology (NIOO‐KNAW) Wageningen The Netherlands; ^18^ Leiden Centre for Applied Bioscience University of Applied Sciences Leiden Leiden The Netherlands; ^19^ Institute for Biological Physics University of Cologne Cologne Germany; ^20^ Department of Bionanoscience, Kavli Institute of Nanoscience TU Delft Delft The Netherlands; ^21^ Department Molecular Biology Umeå University Umeå Sweden; ^22^ Theoretical Biology and Bioinformatics, Department of Biology Utrecht University Utrecht The Netherlands; ^23^ Institute de Biologie, École Normale Supérieure, CNRS, Inserm Paris France; ^24^ University of Bristol Bristol UK; ^25^ Department of Microbial Population Biology Max Planck Institute for Evolutionary Biology Plön Germany; ^26^ Laboratoire Biophysique et Évolution, CBI, ESPCI Paris, Université PSL, CNRS Paris France; ^27^ Institute of Biology, Leiden University Leiden The Netherlands; ^28^ AMOLF Amsterdam The Netherlands; ^29^ Université Paris Cité, IAME, UMR 1137, INSERM Paris France; ^30^ Institut de Biologie de l'Ecole Normale Supérieure Paris France; ^31^ Laboratory of Genetics Wageningen University & Research Wageningen The Netherlands; ^32^ Department of Animal Ecology Netherlands Institute of Ecology (NIOO‐KNAW) Wageningen The Netherlands; ^33^ Informatics Institute University of Amsterdam Amsterdam The Netherlands; ^34^ Department of Zoology University of Oxford Oxford UK; ^35^ Groningen Institute for Evolutionary Life Sciences University of Groningen Groningen The Netherlands; ^36^ San Francisco State University San Francisco California USA

**Keywords:** disease modelling, evolution, evolutionary control, models, population genetics, predictability, prediction

## Abstract

Evolution has traditionally been a historical and descriptive science, and predicting future evolutionary processes has long been considered impossible. However, evolutionary predictions are increasingly being developed and used in medicine, agriculture, biotechnology and conservation biology. Evolutionary predictions may be used for different purposes, such as to prepare for the future, to try and change the course of evolution or to determine how well we understand evolutionary processes. Similarly, the exact aspect of the evolved population that we want to predict may also differ. For example, we could try to predict which genotype will dominate, the fitness of the population or the extinction probability of a population. In addition, there are many uses of evolutionary predictions that may not always be recognized as such. The main goal of this review is to increase awareness of methods and data in different research fields by showing the breadth of situations in which evolutionary predictions are made. We describe how diverse evolutionary predictions share a common structure described by the predictive scope, time scale and precision. Then, by using examples ranging from SARS‐CoV2 and influenza to CRISPR‐based gene drives and sustainable product formation in biotechnology, we discuss the methods for predicting evolution, the factors that affect predictability and how predictions can be used to prevent evolution in undesirable directions or to promote beneficial evolution (i.e. evolutionary control). We hope that this review will stimulate collaboration between fields by establishing a common language for evolutionary predictions.

## 1 INTRODUCTION

Important questions for battling diseases, improving biotechnology and protecting biodiversity include the following: ‘Which pathogen strains will be most prevalent a month from now?’, ‘When and where will pathogenic mutants that escape vaccine‐conferred immunity arise?’, ‘Which patient will be cured of cancer and which patient will see the tumour come back, but now resistant to chemotherapy?’, ‘Can we use gene drive systems to get rid of (vectors for) dangerous diseases or will they evolve resistance to the gene drive?’, ‘How fast will a strain engineered for ethanol production evolve and lose its efficiency during prolonged fermentation?’ and ‘Which endangered species will go extinct and which will adapt successfully to their changing environment?’.

Answering these questions requires the ability to predict the future course of evolution. In addition, some of these situations would have us trying to influence the course of evolution. While some fields have been working for many years on predicting and influencing evolution, for other fields this is a new endeavour. We argue that predicting and trying to influence evolution is more common than one may think, but it is not always easy to recognize because the jargon used in different fields is varied. The main goal of this review is to show the breadth of situations in which evolutionary predictions are being made. In addition, we aim to provide a common language to improve information transfer between research communities. We discuss the study of the predictability of evolution, we describe different methods for evolutionary forecasting and we discuss situations where the goal is to influence evolution (evolutionary control).

Note that throughout this paper, we focus on predictions of how populations will evolve, that is how the genetic and phenotypic makeup of populations will be different in the future (Nosil et al., 2020), rather than predictions about the evolution of new species, or predictions about the past. We thus generally take a more applied approach to predict evolution compared with some of the existing literature (Conway Morris, 2003; Gould, 1990).

### 1.1 The scientific basis of evolutionary predictions

What is the basis upon which we can make sound predictions about evolution? Evolving populations are complex dynamical systems and one has to take into account different forces (e.g. directional selection), including stochastic effects (mutation, environment) and nonlinear dynamics (e.g. due to eco‐evolutionary feedback loops). Evolutionary predictions are often based on Darwin's theory of evolution by natural selection, which states that if populations of entities manifest heritable variance in traits (that contribute to fitness), then these populations will adapt to their environment. For example, we can predict that if we treat bacteria with antibiotics, and if these bacteria harbour (or acquire) genetic variation for antibiotic susceptibility then the bacterial population will adapt to that challenge and become resistant. We can also recreate this scenario as an experiment in the lab and see whether our prediction holds true.

Some extensions to Darwin's theory make these statements more precise and quantitative. For example, our understanding of the polygenic nature of quantitative traits has aided in developing tools such as the ‘breeder's equation’ and ‘genomic selection’, facilitating selective breeding strategies in order to deliver particular (predicted) outcomes in animal husbandry and agriculture (Cooper et al., 2014; Masuka et al., 2017). For other situations, we need more explicit population genetic models to include forces that can distort the expected impact of selection, such as random genetic drift, migration, recombination and mutation, and the stochasticity associated with these forces.

An additional complicating factor is that populations impact their environment. In many situations, we therefore have to consider both evolutionary and ecological dynamics and these can feedback onto each other (eco‐evolutionary feedback loops). For example, the fate of endangered species may critically rely on the abundance of predators, preys and other members of their ecological community, while these populations are in turn affected by the endangered species in question (Govaert et al., 2019).

Predicting evolution has long been considered challenging or even impossible. Fundamental difficulties of predicting evolution include the inherent stochasticity of mutation, reproduction and environment, and the unknowns of the genotype‐phenotype and phenotype‐fitness maps which, together, determine the fitness landscape (De Visser & Krug, 2014; Fragata et al., 2019; Wright, 1932). In addition, eco‐evolutionary feedback loops make long‐term predictions challenging. These aspects of evolving populations will limit the accuracy, and predictions will therefore always be probabilistic and provisional, especially for predictions further into the future. Thus, short‐term and microevolutionary predictions may be most achievable (Lässig et al., 2017).

### 1.2 Why predict evolution?

**FIGURE 1:**
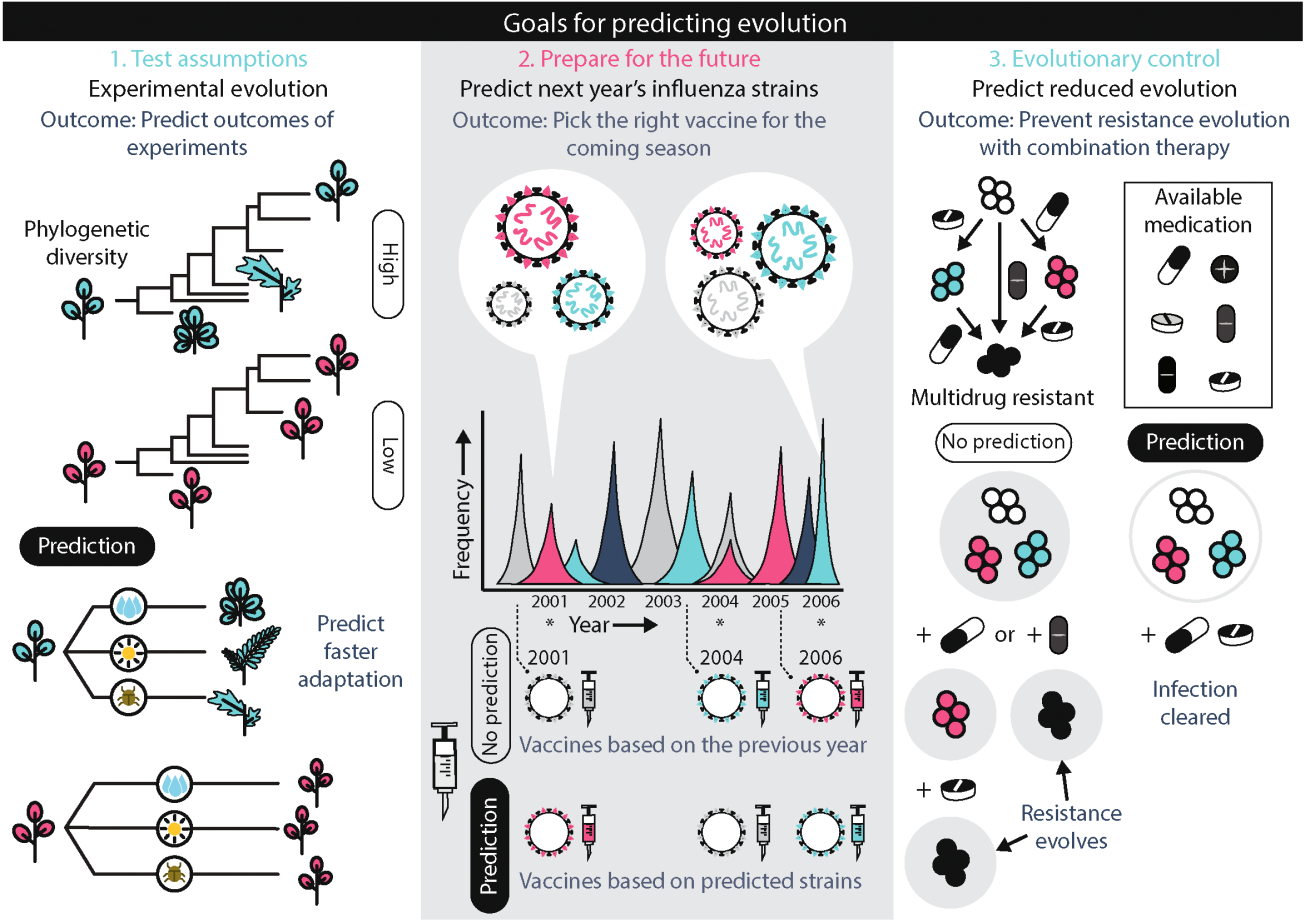
Why do we need predictions? (1) To test hypotheses of evolution for a better fundamental understanding of evolving systems. Based on their phylogenetic history we can predict how species evolve when exposed to a given treatment. These predictions can be tested with experimental evolution approaches. (2) To be prepared for future outbreaks, we aim to match vaccines with the most common influenza strains each year. (3) To have control over evolutionary outcomes and design treatment strategies that prevent the evolution of resistance from happening in pathogens. In this review, we focus on predicting evolution for goals (2) and (3), while (1) plays a role in obtaining the information on the basis of these predictions.

There are different reasons why we are interested in predicting evolution, which we have organized into three main categories (Figure 1). In the first category, although not the focus of this paper, are predictions that are used for experimental systems to develop fundamental knowledge on evolving systems and to test assumptions of models that are used to predict future evolution (Figure 1a). Most work in experimental evolution falls into this category. These experiments can focus on the speed of adaptation, the distribution of fitness effects of new and existing mutations, the repeatability of evolutionary outcomes and the causes of such repeatability. Several studies using experimental evolution with *E. coli* have revealed general rules of microbial adaptation. For instance, (i) fitness improvement is faster in maladapted genotypes (Couce & Tenaillon, 2015), (ii) the beneficial mutation supply is large, such that often multiple beneficial mutations coexist and compete in a population (Lang et al., 2013), (iii) in most environments mutations with large fitness benefits are only found in a few genes (Lind et al., 2017; Tenaillon et al., 2012), which leads to high evolutionary convergence at the gene level, (iv) mutations with large fitness benefits typically occur at a low rate (Schenk et al., 2022) and (v) a change in mutation rate can easily be selected for in the course of adaptation (Sniegowski et al., 1997). These observations, while made mostly in vitro, were recovered in (experiments in) more natural conditions such as the mammalian gut (Barroso‐Batista et al., 2014; Lescat et al., 2017; Zhao et al., 2019). Experiments to test fundamental knowledge and assumptions force us to define the necessary information to predict the evolution and determine reasons for failure, and they allow us to test the limits of the generality of predictions.

A second reason for making evolutionary predictions is to be prepared for the future. A key example here is seasonal influenza (Figure 1b). In the spring of any year, vaccines are produced for the next fall. To make sure the vaccine is as effective as possible, it is necessary to predict which strains will be most common in the next influenza season.

A third reason to predict the course of evolution is to choose actions that influence the direction or speed of evolution—also referred to as evolutionary control (Figure 1c). Evolutionary control is the alteration of an evolutionary process with a specific purpose. Control can either suppress evolution, for example, prevent pathogens from evolving drug resistance, or facilitate evolution, for example, increase the ecological range of a species to avoid extinction. As an example of the first, treatment regimes may be chosen with different combinations of antibiotics that, together, reduce the risk of resistance evolution or that guide evolution to low fitness types that are less likely to spread in antibiotic‐free environments. There are general measures to achieve these goals, but measures can be more targeted if we can predict their effects on evolution. We devote a section of this review to evolutionary control.

For predictions in the second and third categories above, it might be enough to just predict the future without knowing why a prediction is correct, for example, by using machine‐learning or other statistical methods. In other words, a useful prediction does not always come from an understanding of the underlying mechanisms. However, precise predictions often come at the expense of generality, which means predictions cannot be applied in conditions that are even slightly different (Huneman, 2014). This is related to Levins' triangle, following the 1966 paper in which Levins states that models cannot simultaneously achieve realism, precision and generality (Levins, 1966).

### 1.3 *What* do we want to predict?

When making evolutionary predictions, we can focus on many different aspects of the future state of a population. Here it may be useful to briefly compare evolutionary predictions to weather and climate predictions, which have many dimensions as well. Sometimes we care about *whether or not* it will rain tomorrow; whereas other times, when we worry about flooding, we care about exactly *how much* rain will fall in the next 24 h. Also, on most days, wind speeds may not be mentioned in a weather forecast, but when a hurricane is arriving, wind speeds are suddenly crucial to prepare for the impact of the hurricane. Evolutionary predictions are similarly diverse. Evolutionary predictions can be about different population variables (e.g. majority phenotype or genotype, average fitness, identity of fixed mutations, allele frequencies, population size) and include a time component (from a few hours to many years).

In this paper, we focus on evolutionary predictions that are forward‐looking in the sense that they concern future events. Predictions can be focussed on either the genotypic or the phenotypic level. For instance, at the genotypic level, we can predict the frequency of influenza variants in the next influenza season and *which strains* will be most common (Łuksza & Lässig, 2014), or we can predict the mutational targets of *E. coli* responding to various environmental pressures (Wang et al., 2018). At the phenotypic level, we can predict the shape of Darwin's finches' beaks after a drought (Grant & Grant, 2002). Other times, we want to predict *whether* or *after how long* drug resistance will evolve in a virus or bacterial infection in a patient. In experimental evolution, the goal may be to predict a certain *phenotype* (e.g. cell size) or the *average fitness* of a population after some amount of time, or we may want to predict *which genes* will acquire new mutations or confer increased fitness. With increasing interest in engineering microbial communities, interest turns to predict the evolution of *interacting populations*. In conservation biology, the focus may be on predicting future *population sizes*. For all of these cases, besides predicting the most likely outcome, sometimes the *probability* of a *certain outcome* (e.g. extinction) is most important.

### 1.4 A conceptual model of evolutionary predictions

Although the objects of evolutionary predictions are highly diverse, at an abstract level, they nevertheless share a common structure (Figure S1). All evolutionary predictions result from a model—including conceptual, verbal, mechanistic, statistical, computational or mathematical models—that allows for a projection of the state of the evolving system beyond the input that is provided.

The model for all evolutionary predictions starts from describing the current state of the evolving system and incorporates prior scientific knowledge of relevance (e.g. facts and mechanisms, evolutionary processes, etc.). This is the input of the model. The assumptions that are made, and that have not (yet) been proven to be true can also form input or constraints for the model. The output of the model describes the state of the evolving system in the future.

When we describe predictions, we can consider various attributes. First, we should consider which attributes of a population we want to predict, or what is the predictive scope: Is the prediction about genotypes, phenotypes, fitness or population sizes? Are we trying to predict the average feature of a population or the distribution of a trait? Are we predicting the evolutionary path or the outcome?

Second, we need to consider the temporal range of a prediction, the predictive horizon. Generally, predictions of a system's trajectory are more precise for the near future and lose reliability on a characteristic scale. This is relevant especially when predictions are needed to decide on actions, such as which vaccine to manufacture. For example, when predicting influenza strain frequencies, predictions are useful for up to about one year into the future but not for longer periods (Lässig et al., 2017).

Third, we can consider the level of detail of a prediction or predictive precision. A prediction about the direction of an effect is less detailed than one that also includes its magnitude, rate or trajectory. For instance, predicting that microbes will evolve to consume a novel food source is less detailed than predicting that the evolution will occur via a given sequence of mutations (trajectory) (Lind et al., 2019). In a sense, predictions are a type of hypothesis, and if they are too general, they cannot be falsified.

The fourth attribute of a prediction may be the *a priori* likelihood (absence of ‘surprisingness’) of the prediction, the predictive risk. All else being equal, predicting something which is a priori unlikely, given background knowledge, is harder to predict but possibly more interesting.

## 2 WHAT MAKES EVOLUTION MORE OR LESS PREDICTABLE?

Most of this paper focuses on studies that *make* evolutionary predictions, but to predict evolution, it has to be predictable in the first place. The study of predictability and repeatability of evolution is of wide interest and we'll provide a short discussion of the main issues here (Chevin et al., 2022; De Visser & Krug, 2014; Imhof & Schlötterer, 2006; Miton & Tokuriki, 2016; Rego‐Costa et al., 2018; Szendro et al., 2013). Many factors influence the predictability of evolution. In a highly predictable scenario, the efficiency of selection is high relative to the stochasticity of genetic drift, mutation, recombination and unpredictable environmental changes. In such a situation, the fitness increase of the population and possibly the increase in population size, and key fitness‐related traits can be accurately predicted with deterministic models. An example is described by Feder, Pennings, and Petrov (2021) who show that in 19 of 20 patients infected with HIV and treated with a single drug (3TC), viral drug resistance was fixed within 3 months through exactly the same mutation (M184V) in the reverse transcriptase gene. In this exceptional case the genotype, the phenotype and the timing of evolution are predictable.

There are many reasons why the predictability of one or more aspects of evolution is usually much lower: if the mutation supply is low then waiting times for a successful beneficial mutation will be stochastic. Fitness effects may be influenced by interactions with other mutations (linkage, epistasis and pleiotropic effects) and the environment (and thus selection coefficients) may change in unpredictable ways. Predictability may also be affected by the mating system, recombination (or lack thereof), species interactions, feedback loops and historical contingency. We will not discuss all of these in detail but address a selection of the genetic and ecological factors that affect the predictability of evolution.

### 2.1 Genetic factors

Many genetic factors can influence the predictability of evolution, and here, we discuss three of them, namely mutation bias, mutational supply and epistasis.

We first consider the influence of mutation bias and the distribution of fitness effects on the predictability of evolution. Mutation bias describes the variability in mutation rates of different mutation classes (e.g. transitions vs. transversions) or genomic sites. The distribution of fitness effects (DFE) of new mutations tells us what percentage of mutations have what fitness effect. Variation in mutation rate (mutation bias) and fitness effects of mutations (selection bias, DFE) can both enhance parallel evolution (and hence predictability) by reducing the number of ‘successful’ mutations that achieve fixation or high frequency (Stoltzfus & Yampolsky, 2009; Storz, 2016; Storz et al., 2019). These successful mutations are either favoured by the existing mutation bias (i.e. they occur at a higher rate than other mutational classes), or they provide the largest benefits (Gerrish & Lenski, 1998; Schenk et al., 2022).

Theory predicts that in the absence of selection (i.e. under neutrality), mutation bias is the only driver of parallel evolution (Kimura, 1983). But even when selection occurs, a strong mutational bias reduces the spectrum of mutations available for selection and should therefore increase predictability. Somewhat counter‐intuitively, when selection is very strong for multiple possible mutations, mutation bias is again as important as it is under neutrality (Stoltzfus, 2021). In a wide range of taxa, mutation bias explains a non‐negligible proportion of cases of parallel genetic evolution (Bailey et al., 2017, 2018; Stern & Orgogozo, 2008; Stoltzfus & McCandlish, 2017). For instance, an elegant study on adaptation to high altitudes in birds found parallel evolution, in part due to mutation bias at CpG sites (Storz et al., 2019). When highly beneficial mutations are under‐sampled due to the existing mutational bias, other smaller effect but more frequent mutations may fix instead. Such a pattern was observed in replicated evolving populations of bacteriophage (Sackman et al., 2017) where the mutation with the largest fitness effect was not the one that reached fixation most often, because its mutation rate was lower than that of other mutations with smaller fitness effects.

The impact of selection bias (i.e. fitness effect of different mutations) can be analysed via the distribution of fitness effects (DFE). The above‐mentioned bacteriophage study (Sackman et al., 2017) experimentally quantified the fitness effects of new mutations and then used the shape of the quantified DFE and number of beneficial mutations to predict the probability of parallel evolution (eqn 37 in Joyce et al., 2008), comparing those estimates to observed measures of parallel evolution within the same system. The authors found that including the shape parameters of the DFE in a model improved estimates of the probability of parallel evolution, providing support for the idea that DFEs are important drivers of evolutionary predictability. On the other hand, theoretical work using extreme value theory has shown that regardless of the specific shape of the entire DFE (i.e. including deleterious, neutral and beneficial mutations), there will always be much more small than large‐effect mutations. This can reduce the predictability of which mutation will fix or how fitness will increase because the more numerous small‐effect mutations may collectively have a similar fixation probability compared with the small set of large‐effect mutations (Joyce et al., 2008; Sackman et al., 2017).

Mutational supply is the total number of mutations that occur in a generation (or other unit of time) within a population and, hence, is determined by the population size and the mutation rate. When the mutational supply is low (e.g. in small populations), then having only a few large‐effect beneficial mutations means that the waiting time for one of these mutations may be long, making the timing of their appearance unpredictable (Orr, 2005). With an increase in the mutation supply rate, selection bias becomes the dominant driver of adaptive trajectories, though mutation bias still has an impact on the identity of successful mutations. For instance, in larger populations where mutational supply is high, multiple beneficial alleles are present simultaneously (i.e. the clonal interference regime). Here, selection bias is expected to dominate over mutation bias and genetic drift, and fix the most beneficial mutations largely independent of their mutation rate (Bailey et al., 2017; Pennings et al., 2022; Pinheiro et al., 2021; Szendro et al., 2013).

Another important factor influencing predictability is interactions between mutations (epistasis), which introduce ruggedness in fitness landscapes. Generally speaking, epistasis reduces predictability, because even if fitness effects are measured in one genetic background, we do not know the effects in another background (Miton & Tokuriki, 2016). Additionally, the complexity and redundancy in genotype‐phenotype maps decrease the predictability of evolution: if many different genotypes map to the same phenotype, the observation of any particular genotype is just one of many, equally probable, evolutionary outcomes (Zheng et al., 2019). However, there are interesting nuances; for example, when epistatic interactions change the sign of mutational effects from beneficial to deleterious or vice versa, a condition referred to as sign epistasis (Weinreich et al., 2005). Sign epistasis can both increase and decrease predictability. A seminal study on the antibiotic resistance enzyme TEM‐1 β‐lactamase showed that sign epistasis can strongly reduce the number of mutational pathways along which a population can evolve towards higher fitness (Weinreich et al., 2006), which increases the predictability of evolutionary trajectories. On the other hand, sign epistasis can also lead to fitness landscapes with multiple peaks (Poelwijk et al., 2011), which means that populations can end up moving towards different fitness peaks depending on which mutation fixes first, thus decreasing the predictability of evolution.

How the combined knowledge on these genetic factors aids us towards forecasting organisms' responses to changing conditions in the future is illustrated in the predictability of the mutational routes for adaptive ‘wrinkly spreader’ phenotypes of *Pseudomonas* (Lind et al., 2019). *Pseudomonas* can evolve to grow flattened or wrinkled colonies that compete for access to oxygen. Three mutational routes have commonly been found to underlie the convergent evolution of the wrinkly spreader phenotype (McDonald et al., 2009). However, a study that eliminated these three mutational routes revealed 13 other routes that also led to the wrinkly spreader phenotype. These other paths had similar fitness, but much lower mutation rates (e.g. because of smaller mutational target size), which explained why they were not observed in the original studies (Lind et al., 2015). This detailed information on mutational biases that affect the genotype‐phenotype map could then be used to forecast genetic evolution for wrinkly spreader phenotypes in other *Pseudomonas* species (Pentz & Lind, 2021). Detailed knowledge of the genotype‐phenotype map may seem superfluous when phenotypic evolution can be forecasted without this knowledge, as in the case of the wrinkly spreader phenotype in *Pseudomonas*. However, knowledge of the genetics constraining evolutionary responses is relevant when we want to use evolutionary forecasting to control populations.

Genetic evolution of many traits, especially complex traits in eukaryotes, is highly polygenic, i.e. many recombining loci across the genome with small phenotypic effects jointly contribute to the evolutionary response (Boyle et al., 2017; Pritchard et al., 2010). Polygenic evolution is less predictable in terms of which mutation will fix due to genetic redundancy (Yeaman, 2022). Under genetic redundancy, similar phenotype and fitness outcomes can be achieved through different genetic changes; however, these changes may be more predictable when causal genes cluster in narrow genomic regions or when ancestral genetic variation is repeatedly used in evolution (Blankers et al., 2019; Conte et al., 2015; Tennessen & Akey, 2011; Yeaman, 2022). Polygenic forecasting approaches that focus on phenotypic outcomes use quantitative genetics frameworks that predict the response to selection based on phenotypic and genetic variances and covariances, i.e. the breeder's equation (Lande & Arnold, 1983), or trait‐fitness covariance (Price, 1970; Robertson, 1966) also known as the Robertson‐Price identity. Despite their simplifying assumptions, approaches using quantitative genetics can be successful in forecasting evolution over short time frames (Hill & Kirkpatrick, 2010; Morrissey et al., 2010). Limitations of these approaches are the inability to account for drift effects (Pélabon et al., 2021) and the assumption of linear genotype‐phenotype mapping (Milocco & Salazar‐Ciudad, 2020). A formidable challenge is thus to integrate stochasticity and nonlinearities in frameworks that are amenable to forecasting polygenic evolution (Milocco & Salazar‐Ciudad, 2022).

### 2.2 Ecological factors

Experimental evolution studies are typically performed in a laboratory environment where most environmental and ecological conditions are controlled. However, the predictability of evolution in the wild will depend on the characteristics of populations and their habitat as well as interactions with the biotic and abiotic environment (here jointly referred to as ‘ecological factors’). Ecological factors affect the predictability of evolution both through their effect on the amount and distribution of genetic variation and on the fitness effects of variants. To illustrate this, below, we briefly outline the effect of the rate of environmental change, the characteristics and complexity of the habitat, and the ecological interactions within a community on the predictability of evolution.

Firstly, the speed of environmental change may affect predictability by setting the strength and variability of selection pressures. Overall, adaptation is more likely under gradually changing environments compared with rapid or saltational environmental shifts (Bell & Gonzalez, 2011; Radchuk et al., 2019). Empirical evidence for the higher predictability during gradual change was provided with a yeast laboratory system exposed to different gradients of salt stress (Bell & Gonzalez, 2011). Also, a recent study emphasized that current global climate change causes imperfect adaptive responses due to the high speed of environmental change (Radchuk et al., 2019). An exception is when the change is so fast that populations cannot cope, in which case the predictability of the evolutionary outcome—local extinction—is high. Fast environmental changes impose large selection pressures, which can increase predictability (Gorter et al., 2017), but when the environmental change exceeds the limits of extant spatio‐temporal variation in the habitat, the new fitness landscape becomes largely unexplored. This indicates that fast‐changing environments can limit the ability to predict evolution. Moreover, when environmental change is accompanied by the erratic occurrences of extreme conditions that dramatically alter the fitness landscape, either temporally or spatially, it introduces high stochasticity, further reducing predictability.

Secondly, characteristics of a species' habitat can also affect predictability, including the complexity or heterogeneity of the habitat, patch size and connectedness. The more complex a habitat, the more selective pressures act, which may reduce the predictability of evolution. This can arise through trade‐offs between traits for adaptation to multiple selection pressures (Armbruster et al., 2014; Roff & Fairbairn, 2012; Stuart et al., 2017; Svensson et al., 2021), as well as by decreases in population size. Patch size strongly influences population size, and the connectedness of habitat patches will influence the flow of individuals and, as a consequence, the influx of genetic variation. Dispersal between populations has a dual influence on adaptation: (1) Local dispersal may provide a genetic and/or demographic rescue effect, by effectively increasing the population size resulting in less drift and a higher absolute input of mutations (but see Szendro et al., 2013). (2) High dispersal rates may, however, induce a migration load between populations adapting to different stressors (Bisschop et al., 2019).

Finally, feedbacks between ecological and evolutionary dynamics (eco‐evo dynamics) will influence evolutionary predictability. We know that higher ecological complexity (more diversity, more interactions) promotes ecological stability in some cases (Ives & Carpenter, 2007; Pennekamp et al., 2018; Xu et al., 2021). Can we expect evolutionary dynamics in more complex communities also to be more predictable? One may expect that it is more straightforward to predict evolutionary outcomes in simple communities because there are fewer parameters to take into account. However, this argument is based on data constraints and not on fundamental constraints to evolutionary predictions in complex communities, where feedbacks might stabilize communities, resulting in more stable short and long‐term dynamics. Ecological feedback may lead to frequency‐dependent selection in various ecological interactions. In particular, negative frequency‐dependent selection (NFDS) can lead to predictable frequency fluctuations and stable equilibria of polymorphisms within a population. The strength of NFDS and the (un)predictability of environmental changes then determine whether this leads to unpredictable chaos or whether it can increase the predictability of evolution (Chevin et al., 2022). Moreover, when environmental variability strongly affects population growth and natural selection, as is commonly observed in natural systems, this ‘environmental forcing’ tends to render the evolutionary responses and tracking of the environment less chaotic and more predictable (Rego‐Costa et al., 2018). Therefore, although the complexity of the real world and the inherent stochastic nature of some core ecological processes (such as priority effects (Fukami, 2015)) suggest limits to our ability to predict evolutionary change, exploring the effect of eco‐evo dynamics on the stability of dynamics across timescales can shed new light on the potential for evolutionary forecasting.

Empirical support for the effect of ecological factors on the predictability of evolution exists. For example, in a microcosm experiment with *Escherichia coli*, the spread of beneficial genotypes was mostly stochastic in communities with low complexity but deterministic in high‐complexity communities (Imhof & Schlötterer, 2006), suggesting a positive relationship between ecosystem complexity and predictability of evolution. Similarly, communities of bacteria that were experimentally evolved for several hundred generations followed repeatable trajectories towards a final, stable community structure (Celiker & Gore, 2014). Natural ‘laboratories’ also offer insight into the role of ecology on evolutionary predictability, for example in the form of parallel evolution in replicated habitats where organisms respond similarly to similar changes in their environments. Urbanization provides a unique setting where similar environmental changes are replicated amongst cities, such as the selection of a behavioural gene in the common blackbird (*Turdus merula*; Donihue & Lambert, 2015; Mueller et al., 2013). Other examples are the convergent evolution of colour morphs in Hawaiian spiders caused by their surrounding environment (Gillespie et al., 2018), and reduced armour when marine three‐spine sticklebacks (*Gasterosteus aculeatus*) colonized freshwater, which is explained by both abiotic and biotic changes (differences in salinity and predation pressure, respectively) (Jones et al., 2012). Based on these patterns of repeated evolution, we can potentially forecast how other populations would respond to similar ecological or environmental factors.

## 3 METHODS FOR PREDICTING EVOLUTION

**FIGURE 2:**
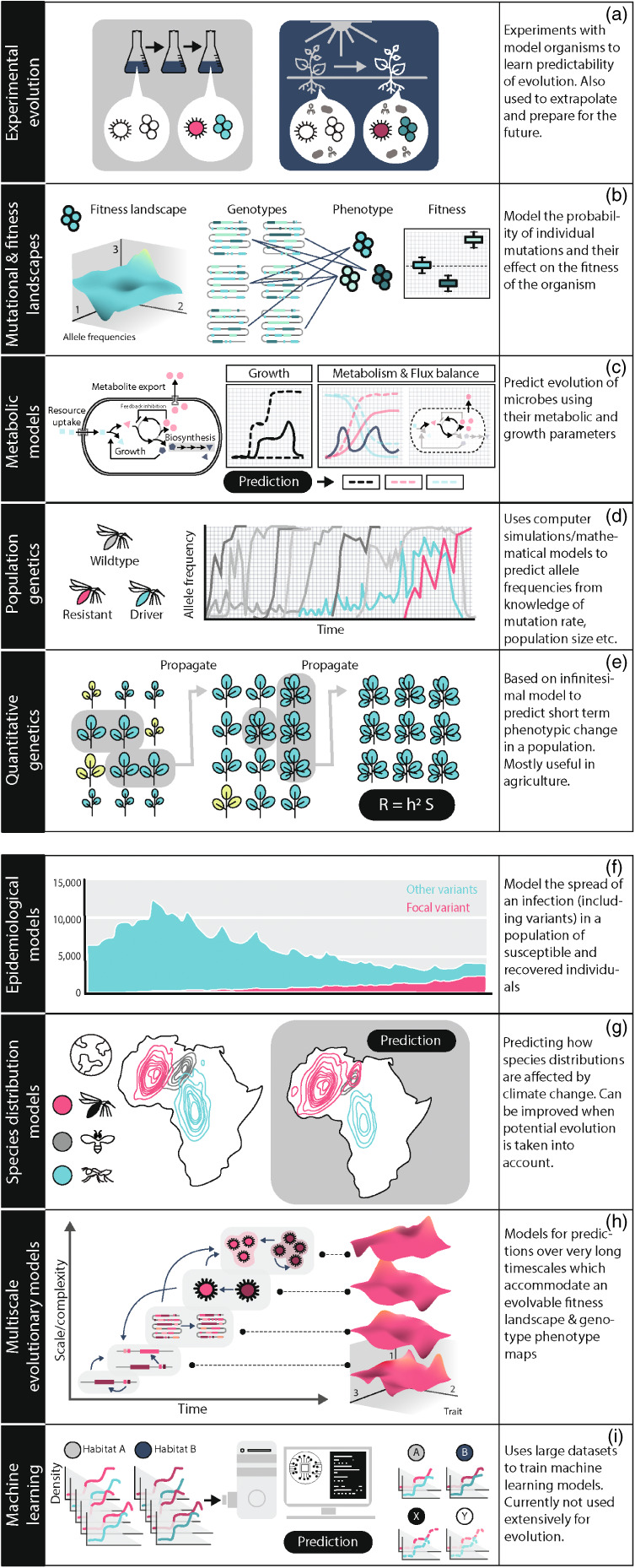
Selection of methods that are used to predict evolution

If we establish that we want to predict evolution, and what such a prediction entails, the next question is how we can predict evolution. Predictions can be data‐driven, e.g. based on observations of repeatability that we would expect to observe again under similar conditions, or based on theory and mechanistic understanding of the evolutionary processes which we can model. Sometimes a combination of the two is used. There are many different approaches and methods to predict evolution. We mention some of those here and in Figure 2. For more details, we refer readers to the Appendix S1. Note that phylogenetic models are not described here but feature in Box 1.

BOX 1 **Examples of evolutionary predictions**

**Evolutionary predictions and control are a guide in the SARS‐CoV‐2 pandemic**

SARS‐CoV‐2, the coronavirus that started a pandemic in late 2019, is a focus of evolutionary predictions at the phenotypic and genetic levels. The extremely high global prevalence of this virus as well as the recent host change and change in selective pressures (such as with vaccines) as well as the unprecedented scale of data that are available make it possible to quickly test the accuracy of many predictions. It is common for viruses to adapt further after a host switch. At first, the main aim of predictions by public health agencies was to predict total case numbers using epidemiological or statistical models with no evolutionary dynamics included (Bertozzi et al., 2020; Watson et al., 2021). Next, there was interest in predicting the dynamics of different strains (Davies et al., 2021). And when vaccines became widespread, the big question was whether and when immune escape would happen (Kustin et al., 2021).

In the absence of herd immunity (due to infections or vaccines) the strongest selection is expected to be on increased transmission, whereas weaker selection is predicted on other disease life‐history traits, such as the lengthening of the presymptomatic phase and decreased virulence. When large parts of the population become immune due to infection or vaccination, selection for immune escape is expected to become more important (Cobey et al., 2021; Grubaugh & Cobey, 2021).

There is a strong interest in predicting specific genetic changes that will occur in the SARS‐Cov 2 virus (Maher et al., 2022). Different methods have been used to predict which mutations can contribute to the escape evolution of the virus from existing population immunity. These methods use existing genomic surveillance data (Harvey et al., 2021), deep mutational scanning (Starr et al., 2020) and comparisons with different coronaviruses (Armijos‐Jaramillo et al., 2020). Recently, a multi‐strain fitness model has been developed to predict selection windows shaping the antigenic evolution of SARS‐Cov 2 (Meijers et al., 2022).

Public health measures like physical distancing and vaccination not only reduce the number of infections but also can reduce the evolutionary potential of the virus due to a reduced population size, which could lower the rate at which new strains emerge. At the same time, widespread vaccination will increase selection for immune escape mutants. This means that vaccination campaigns and physical distancing rules can be seen, at least in part, as evolutionary control measures. A particularly interesting discussion about the effects of vaccination roll‐out on viral evolution has been discussed in the context of dose‐sparing strategies (e.g. getting more people a single dose or a half dose of a vaccine as opposed to vaccinating fewer people with two doses). Different epidemiological‐evolutionary models predict that dose‐sparing strategies could speed up or slow down the evolution of immune escape (Cobey et al., 2021; Saad‐Roy et al., 2021).

**Sustainable product formation by microorganisms**

The chemical industry poses a high environmental burden and a sustainable alternative is (bio)chemical compound production by microorganisms. The major challenge for the bio‐based production of chemicals is the evolution of reduced product formation. This evolution happens because compound production diverts resources from growth, therefore inducing a fitness cost. We can use mathematical models of microbial metabolism to predict the evolutionary stability of production strains. For example, we can predict that for mutants where product formation is coupled to biomass production, the evolutionary loss is least likely. Computational techniques that use genome‐scale metabolic models can then predict which gene knock‐outs couple product formation to biomass, which should lead to increased stability of product formation (Du et al., 2018). These predictions were applied for formate production by cyanobacteria and experimental validation showed that with growth coupling, indeed no decrease in product formation occurred within a month, whereas without growth coupling formate production decreased after days (Du et al., 2019).

**Adaptation of natural populations to changing environments**

To plan conservation efforts most efficiently we need to predict which species or populations can adapt to changing conditions and which will be threatened with extinction. These types of predictions are difficult to make, but several approaches are taken to enable the forecasting of future population states. (1) We can apply a certain selection pressure in the lab and observe the adaptive potential (e.g. under size‐selective harvesting (Uusi‐Heikkilä et al., 2015) or higher temperatures (Kellermann et al., 2009; Morgan et al., 2020)). (2) Similarly, species can be transplanted to different habitats and their adaptation monitored ((Colautti & Barrett, 2013) and see (Edwards, 2015) for a review). (3) We can determine if ongoing evolution has already led to adaptive change (e.g. a recent meta‐analysis of phenological adaptation of (mostly) birds to climate change showed that it is unlikely that adaptation will rescue populations (Radchuk et al., 2019), although it is difficult to distinguish between evolutionary and plastic adaptation (Chevin et al., 2010); and contrary to predictions it does not seem that Atlantic Cod has genetically adapted to fishing (Pinsky et al., 2021)). (4) We can use species distribution models along with genomic information (Hoffmann et al., 2015). Predictions for the last type have been made for the dwarf birch (Borrell et al., 2020), oaks (Rellstab et al., 2016) and the yellow warbler (Bay et al., 2018).

Approach (1) is difficult or impossible and time‐consuming for many species and it remains to be seen how well these predictions can be extrapolated to natural systems. The latter problem is reduced in approach (2). Approach (3) can only be applied to changes that are already happening and needs a large amount of long‐term data on individuals. Finally, approach (4) has usually yielded very weak correlations. Therefore, predicting which species may adapt to novel environmental conditions (e.g. rapid climate change) remains a big challenge.

**Influenza vaccine development relies on evolutionary predictions**

One of the best‐known examples of evolutionary predictions is predictions of which influenza strains will be common the next season, as a basis for vaccine development. Two main methods, that can also be combined, are used to make these predictions: molecular properties of the virus and genealogical trees (Morris et al., 2018). The first method uses key phenotypes contributing to viral fitness: protein stability and binding to the human antibodies sum up to the fitness of the virus particle (Łuksza & Lässig, 2014). The second method uses data from recent clinical samples and makes a phylogenetic tree of these strains. The ‘bushy’ parts that have a lot of recent diversification are the expected genotypes that will dominate next year (Neher et al., 2014). These methods are in use for vaccine strain selection. However, success rates are still limited and further improvement would have a large impact on the effectiveness of influenza vaccines. Such improvement can come from a better understanding of the genotype‐phenotype map for virus‐antibody interactions, such that antigenic evolution can be predicted better from sequence data.

### 3.1 Experimental evolution

A straightforward method for evolutionary predictions is creating the conditions of interest (in the lab or in a natural environment), observing (a lack of) evolution and identifying which conditions lead to which outcomes (Jagdish & Nguyen Ba, 2022; Kawecki et al., 2012).

### 3.2 Using the mutational and fitness landscape

Though currently out of reach for most systems, it may become possible to predict the next evolutionary step for a population using detailed knowledge of the mutation and fitness landscape for a population in a given environment (Fragata et al., 2019; Salverda et al., 2011).

### 3.3 Metabolic and growth models

When the selection of microorganisms is due to differential population growth of alternative genotypes, we can use (genome‐scale) metabolic and growth models to predict how these populations will evolve (Schuetz et al., 2007; Wortel et al., 2016). Metabolic models have been used to predict de novo, previously unseen mutations that induce dosage‐dependent antibiotic resistance mutations in *E. coli* (Pinheiro et al., 2021).

### 3.4 Population genetic models

Population genetic models are models that keep track of the genetic status (often at one or a few loci) of an entire population (Hartl, 2020). These models are either mathematical in nature or use computer simulations and can include mutation, fitness, reproduction, recombination and other parameters, with both deterministic and stochastic forces. They are used to predict the success of gene drive systems (Champer et al., 2020; Noble et al., 2017).

### 3.5 Quantitative genetics and the breeder's equation

In situations where the focus is on altering one or a few phenotypes, over short timescales and in relatively controlled environments, quantitative genetics has achieved great success in predicting phenotypic changes (Walsh & Lynch, 2018). Such models are used, for example, in maize breeding programmes (Masuka et al., 2017).

### 3.6 Epidemiological models (SIR models)

SIR models are compartment models where individuals can move from a susceptible (S) to an infectious (I) state and from an infectious to a recovered (R) state. When classical SIR models are combined with the possibility of the pathogen to evolve (e.g. changing virulence or infection probabilities), we can predict the spread of an evolving infectious agent (Gordo et al., 2009) or the evolution of a multi‐strain viral population (Łuksza & Lässig, 2014).

### 3.7 Species distributions across space and environmental conditions

Forecasting biodiversity responses to climate change are generally done through species distribution models. Such models can be combined with genomic data and evolutionary responses to predict adaptation (Bay et al., 2017) and range expansion (Kearney et al., 2009).

### 3.8 Multi‐scale evolutionary modelling

Studying long‐term evolution requires models in which the genotype‐phenotype map itself can evolve. Such models can be used to study genome evolution and evolution of communities. One example of a multi‐scale model is the combination of within‐host and epidemiological levels in a model to study the effect of the HIV latent reservoir (Doekes et al., 2017)

### 3.9 Machine learning

In cases where large amounts of data on repeated evolutionary trajectories in the past are available, machine‐learning approaches are likely to become increasingly important for predicting evolution (Hayati et al., 2020; Schenk et al., 2022).

## 4 EVOLUTIONARY CONTROL

### 4.1 Evolutionary control needs predictions

Using predictions for control requires an extension of the scope of the prediction: we have to predict not only the evolutionary process under natural conditions but also its response to specific control interventions. Important applications of evolutionary control are interventions against evolving human pathogens (Lässig & Mustonen, 2020) and against insects evolving insecticide resistance (Tabashnik et al., 2013). In this section, we will describe several examples of evolutionary control, where predictions are used or could prove beneficial to improve control measures.

### 4.2 Preventing or reversing antibiotic resistance in bacterial pathogens

Increasing rates of antibiotic resistance threaten the efficacy of this mainstay of treatment for bacterial disease. Because the discovery and development of novel antimicrobial agents lag behind the rate of resistance evolution, newer approaches that focus on antimicrobial stewardship have emerged whose aim is to prevent or reverse resistance evolution to existing drugs (Andersson et al., 2020; Nichol et al., 2015; Perron et al., 2015; Read & Woods, 2014). These ideas, which can be implemented for individuals or at the population level in a hospital or agricultural setting, fundamentally rely on an accurate, empirical understanding of antimicrobial resistance evolution and spread. Two broad categories of evolutionary predictions to inform therapeutic decisions can be envisioned: one to avoid a specific outcome and another to promote one.

The case of HIV shows that predictions don't have to be very precise in order to allow some control. When triple‐drug therapy became available for HIV patients in the 1990s, the prediction—based on a mathematical model—was that it would prevent or, at least, slow down the evolution of drug resistance, reducing the progression to AIDS and saving lives. Even though the model that was used wasn't entirely correct, the high‐level predictions held, and many lives were saved (Feder, Harper, et al., 2021; Perelson & Nelson, 1999; Rocheleau et al., 2018).

Several different models and experiments have supported the intuitive predictions that combination therapies decrease the likelihood of resistance evolution compared with monotherapy. First, combination therapies increase the rate of pathogen decline, limiting the time window for de novo resistance mutations to occur (Raymond, 2019). Second, combination therapies require resistance mutations in multiple targets, decreasing the probability that completely resistant mutants will arise ((Perelson & Nelson, 1999), though see (Feder, Harper, et al., 2021)). Combination therapies encompass a wide range of approaches: using multiple antimicrobial agents simultaneously (e.g. antibiotic‐antibiotic, antibiotic‐adjuvant, antibiotic‐phage or phage cocktails); antibiotic mixing, that is, random allocation of different antibiotics for different patients in the same hospital ward; and implementing temporally alternating therapies including antibiotic cycling (population level) and sequential therapy (individual level) (Abel Zur Wiesch et al., 2014; Nichol et al., 2015; Sarraf‐Yazdi et al., 2012; Tyers & Wright, 2019; Yen & Papin, 2017).

More interesting are approaches that would be used to drive a particular outcome. These are based largely on known epistatic or pleiotropic effects of resistance mutations. Resistance mutations are typically associated with fitness costs, and several authors have promoted treatment strategies designed to maximize these costs, thus maximizing the probability that these strains are replaced once the antibiotic selection is relaxed (Andersson & Hughes, 2010). Interactions between resistance mutations can also be used to exploit drug synergies, thereby driving faster rates of population decline in the pathogen. Finally, a recent strategy is based on the idea that resistance to a given drug pleiotropically increases susceptibility (i.e. causes collateral sensitivity) to a second drug (Sommer et al., 2017). Knowledge of collateral sensitivity could help choose drugs to be used sequentially: if resistance to one drug evolves, it concomitantly increases the efficacy of the other one. Note, however, that epistatic effects (when a mutation's effect depends on the genetic background) can make it harder to predict, and thus use, collateral sensitivity (Barbosa et al., 2019; Hernando‐Amado et al., 2020).

### 4.3 Insect resistance to transgenic plants

Evolutionary predictions have been used to guide the deployment of the most successful transgenic plants designed to protect crops against insect damage. Cotton, maize and other crops have been transformed with the genes for insecticidal protein toxins from the bacterium *Bacillus thuringiensis* (Bt) and have been remarkably successful as an alternative to sprays with chemical insecticides (Tabashnik et al., 2013). From the beginning, the evolution of insect resistance to Bt toxins was anticipated and deployment strategies were deliberately designed to minimize its spread. For example, toxins were chosen for which resistance was known to be recessive, and for which standing genetic variation for resistance was shown to be low. This was done because population genetic models predict that evolution will proceed more slowly for recessive alleles or when alleles are rare. Population genetic models of natural selection were also used to predict how soon resistance would evolve, based on assumptions about the genetics of resistance, the strength of selection, pest dispersal amongst Bt and non‐Bt crops, and other genetic and ecological factors (Roush et al., 1998). Strategies based on these predictions, especially high dose/refuge strategies, were mandated by governmental regulatory agencies (Meihls et al., 2008). The high dose/refuge strategy means that (1) there is a refuge (crop that does not express the Bt toxin) which means that even if resistance evolves, very few homozygotes for the resistance allele will be produced, while (2) the dose of the toxin is so high that heterozygotes for the resistance allele will die. Retrospective analysis of the efficacy of these strategies has shown that they were more successful when more of the underlying assumptions were valid in the field, and when grower compliance was high (Tabashnik et al., 2013). This is probably the most successful test of the performance of evolutionary predictions in modern agriculture.

### 4.4 Prevention of resistance to gene drives

Gene drive systems use genetic constructs (currently often CRISPR/Cas9‐based) to force the spread of a trait into a population. These systems are being developed to, one day, change wild populations (e.g. make mosquitoes resistant to malaria), however, the target species can evolve to become resistant to the drive allele. Research is therefore focussed on preventing the evolution of resistance.

Gene drives or gene drive constructs are a special type of segregation distorter that use the CRISPR/Cas9 gene editing technology. Under Mendelian expectations, each gamete has a 50% chance to carry the allele that came from one parent and a 50% chance to carry the allele that came from the other parent. One type of gene drives is called homing gene drives. They use a kind of copy‐paste mechanism that distorts the 50–50 rule and therefore they can be present in many more gametes, which then leads to offspring that inherits the gene drive allele at a rate much greater than 50%. This works as follows: when the gene drive construct is initially present in the heterozygous state in a cell, it can cleave a genomic target site in the chromosome that doesn't carry the construct. This cut then induces the cell to repair the damage by copying the drive sequence into the damaged chromosome. The result is that the cell now has two copies of the gene drive allele. In this way, the gene drive construct can rapidly spread in the germline and therefore in the population. When the gene drive system is linked to an allele of interest (the ‘payload’), the transmission bias forces the spread of that allele. While CRISPR/Cas gene drives are not yet used outside the laboratory, there are plans to use this technology in mosquitoes and other species that cause harm to humans.

Resistance to homing gene drive systems can evolve when the cut that is made by the gene drive construct is repaired by the nonhomologous end‐joining pathway. This is because end‐joining often leads to changes in the target sequence, which means that the drive construct can no longer cleave that sequence (Champer et al., 2020; Gomulkiewicz et al., 2021). When resistance has thus evolved, a drive construct can no longer spread and may go down in frequency if it comes with a fitness cost.

Evolutionary predictions at two different levels are of interest here: (1) how fast will the drive allele spread in the population and (2) when will resistance to the gene drive evolve and spread? The first is a fairly straightforward application of existing population genetic and population dynamic models, with an additional parameter for non‐Mendelian segregation. However, in early experiments, the populations almost always became resistant to the gene drive element. The second level of prediction is therefore possibly more important: how fast will resistance evolve (Dhole et al., 2020; Gomulkiewicz et al., 2021; Unckless et al., 2017)? The susceptibility of a gene drive construct to resistance can be reduced by (a) increasing the number of sites at which the gene drive construct can cut (Champer et al., 2020) or by (b) introducing a cost to resistance by targeting the gene drive to an essential gene (Noble et al., 2017). Predictions have been validated by laboratory experiments: several groups have shown that gene drives with multiple guide RNAs (which target multiple sites), or that target an essential gene, can spread in a population for much longer before resistance evolves (Champer et al., 2020; Kandul et al., 2021; Kyrou et al., 2018). In an important example of successful evolutionary control—at least in the lab—researchers from Imperial College London were able to create a gene drive system that targeted an important fertility gene (*doublesex*) in 2018. With this gene drive system, no resistance evolved and the experimental mosquito populations all went extinct as hoped and predicted (Kyrou et al., 2018).

### 4.5 Preventing extinction by promoting evolution

One case where we want to promote evolution is to rescue a species from extinction. It is widely thought that a lack of genetic variation increases extinction risk. For example, the Tasmanian devil had very low genetic variation and its population size was severely reduced by infectious cancer (Hendricks et al., 2017). At the same time, loss of habitat, loss of dispersal opportunities and decrease in population size can lead to lower genetic variation in a species leading to an ‘extinction vortex’ (Olivieri et al., 2015). The main method of promoting adaptation to a changing environment is therefore by increasing genetic diversity, as genetic diversity has been shown to be beneficial for adaptation and rescue (Agashe, 2009; Agashe et al., 2011; Hughes et al., 2008). Maintaining genetic variation also plays a role when breeding programmes are used to rescue a population (Ebenhard, 1995). Increasing genetic diversity in endangered populations, termed genetic rescue, often used to avoid inbreeding depression, is a promising intervention, but whether it predictably leads to increases in population sizes to prevent extinction remains to be seen (Bell, 2017).

## 5 CONCLUSION AND OUTLOOK

Evolutionary predictions are used in many fields, including infectious disease, biotechnology and conservation biology. In some cases, the use of evolutionary models that include mutations, selection and drift is very explicit (such as in the gene‐drive example or in the influenza vaccine predictions), whereas in other cases evolution may only be implicitly included in population size predictions (such as for predicting extinction risk for endangered species). In this review, we have shown how evolutionary predictions are used in many biological subfields. Because researchers in different subfields use different languages, it is not always obvious that they are, in essence, doing the same thing: predicting the future state of an evolving population. Predictions can be improved when researchers can learn and be inspired by results from other subfields, but for this to happen, we need to use a common language. This review is meant as a start to bridge those research communities. We believe that those who work on preventing unwanted evolution (in biotechnology, agriculture and health) and also those who work on promoting or steering evolution (such as in conservation biology and biotechnology) could benefit from much more extensive communication.

Most of the theory we described in this review was featured in the paragraphs on predictability. This reflects the relatively early stage of the field, where many researchers involved focus on understanding under which conditions evolution is more or less predictable and which factors drive predictability. While this has led to many relevant insights, these theoretical insights are still far away from the applications where predictions are needed. We thus believe that the field could benefit from a stronger connection between theory and applications. Specifically, the efforts that are underway in the area of influenza research (using data and theory to predict influenza strains to design the best vaccine) could be replicated in other situations. For example, models could be made to predict drug resistance in a hospital over the next year, given the current state and parameters such as antibiotic use. Also, evolutionary models could more explicitly be used in other situations, such as to predict which tumours will recur with resistance. By applying evolutionary methods to real‐life situations, we will discover the gaps in our knowledge and contribute to making evolutionary predictions more accurate and useful.

Finally, we expect that the increasing access to genome information and the use of modern statistical techniques, including machine learning, will improve evolutionary predictions in amenable taxa over the next few years. However, there will be a continued need to develop mechanistic models of evolution for various systems, for at least two fundamental reasons: (i) to extrapolate predictions to conditions other than those used to parameterize specific models and (ii) to further our understanding of how evolution works. While the increasing access to high‐resolution phenotype and genotype data make it tempting to include all these details in such models, more coarse‐grained mechanistic models may allow more powerful predictions. We hope that together, improved collaboration, a shared language and new combinations of methods will lead to further maturation of the field, leading to evolutionary predictions becoming mainstream in areas such as infectious disease, conservation biology and biotechnology.

## CONFLICT OF INTEREST

The authors declare that there is no conflict of interest.

## Supporting information


Appendix S1

## Data Availability

Data sharing is not applicable to this article as no datasets were generated or analysed during the current study.
