# Supplementary material for: Towards evolutionary predictions: Current promises and challenges
Source: Evol Appl. 2022 Dec 9;16(1):3–21. doi: 10.1111/eva.13513 (PMC9850016; doi:10.1111/eva.13513)
Supplement: Supplementary file 1 — Appendix S1 [file EVA-16-3-s001.pdf]

Supplementary figures

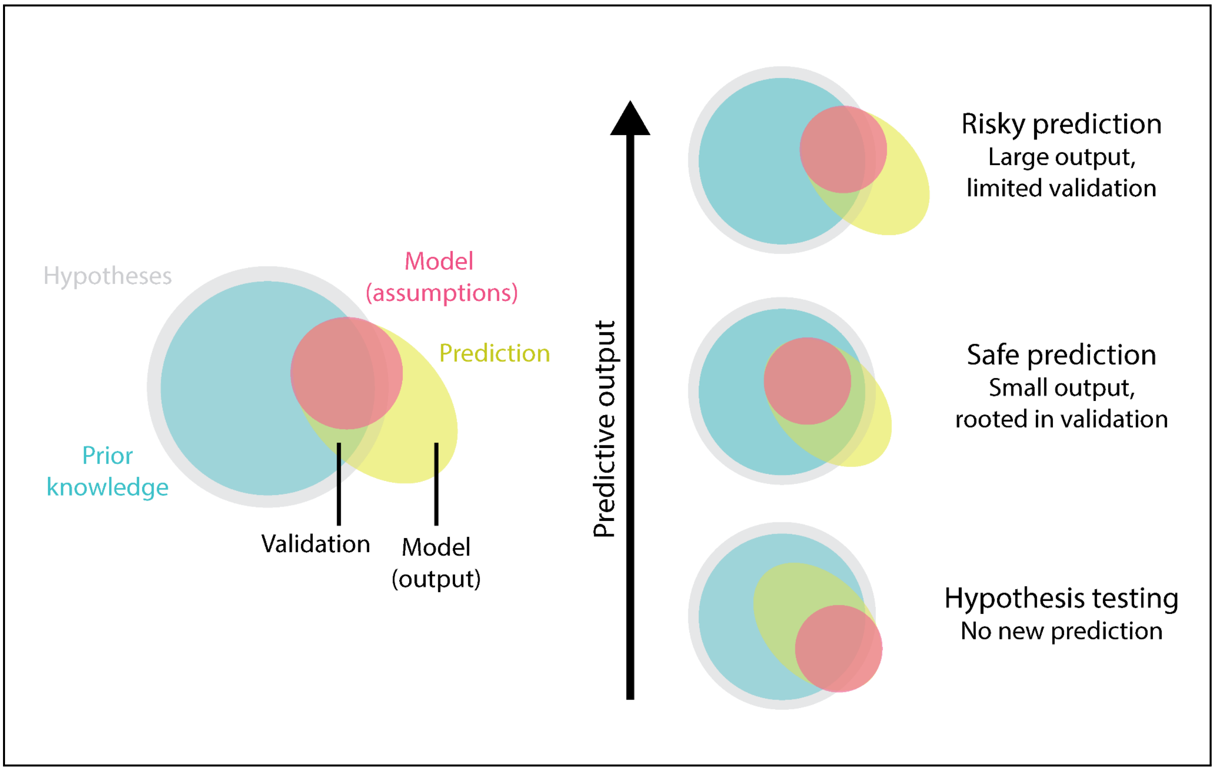

Supplementary Figure 1. Shared abstract structure of all (evolutionary) predictions. *Starting from prior knowledge (blue circle), models can be formalized (pink circle) which may project into the unknown (visualized by the yellow ellipse), using assumptions (grey aura). If pointing outwards, the yellow area of the ellipse constitutes a prediction, whereas the green area is used for validation of the model and its assumptions. The model assumptions and output will determine the orientation of the ellipse, and to what extent it projects into the unknown. The predictive output will range from the most risky predictions of more fundamental and serendipitous nature, to safer predictions which will be more suitable for predicting more practical matters such as potential pathogen escape mutants. Hypothesis testing here is the case where predictions are made about results that are known, to test if the model that we have of a process explains the observations.*

As an example we apply the conceptual model to the prediction about seasonal influenza strains in the next season, which is used to design the next vaccine. Prior knowledge (blue circle) is the current state of the population as inferred by observed frequencies of influenza strains in humans and other hosts, and the trajectory of these frequencies in the recent past. The model assumptions (grey) can include mathematical descriptions of how the frequency trajectories can be extrapolated into the future (taking into account mutation, drift and selection). The outcome of the model (yellow) is the predicted frequencies of the major strains during the next influenza season, only the list of the most prevalent clades, or the antigenic phenotypes of the future virus population.

In terms of the four attributes described in the main text, the predictive scope is the genetic or antigenic composition of the population, the predictive horizon is a time scale 6-12 months into the future. The predictive precision is high in terms of time (we need to know the common strains for the next season, not earlier or later than that) and genetics, as we are predicting the exact strains. The a priori likelihood is bounded when we consider only existing strains, as there may only be a small number of possible outcomes when only few strains currently exist in the population. However, the exact predictive risk depends on whether a common or uncommon strain is predicted to be prevalent the next season. Over longer time scales de novo mutations become important. De novo mutations are hard to predict, which is one reason why predictability is reduced over longer time scales.

Methods for making evolutionary predictions

1. Experimental evolution

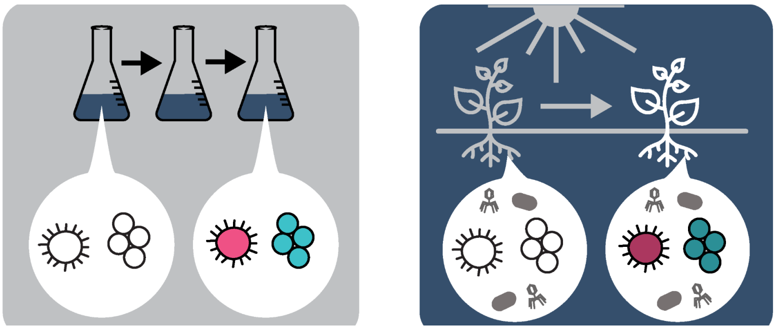

A straightforward method for evolutionary predictions is creating the conditions of interest (in the lab or a different natural environment), observe (a lack of) evolution, and extrapolate from these observations into the future.

Laboratory experimental evolution is diverse; it is commonly used for fast-evolving microbial systems (e.g. antibiotic resistance evolution (Kawecki *et al.* 2012, Remigi *et al.* 2019), but also larger organisms such as *Drosophila* (Burke & Rose 2009) and *Caenorhabditis* (Teotónio *et al.* 2017). Experimental evolution can also include interactions with the environment (i.e. eco-evolutionary interactions), such as the role of environmental spatial structure (Nadell *et al.* 2016) or the natural biotic context (Zandbergen *et al.* 2021).

Field studies are more appropriate for conditions or organisms that cannot be studied in the lab, for example because habitats are too large. In such cases, the effect of environmental conditions on evolution can be studied with reciprocal transplant experiments (Edwards 2015) or observations of “natural field experiments”, such as sticklebacks that have moved from marine environments to fresh water repeatedly (Jones *et al.* 2012).

Experimental evolution can also be used to study the predictability of evolution, and many such studies have indeed focused on the predictability (or repeatability) of evolution (e.g., (Bull & Molineux 2008, Sackman *et al.* 2017, Lind 2019, Schenk *et al.* 2022).

2. Using the mutational and fitness landscape

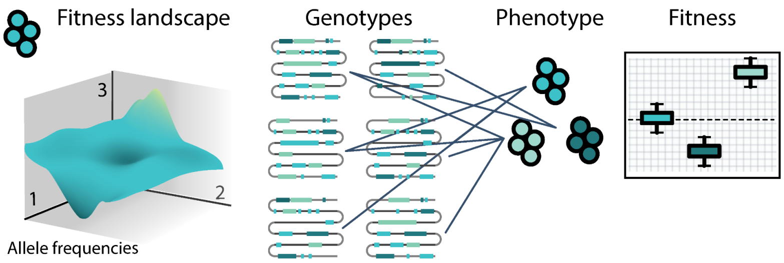

Though currently still out of reach, one day, it may be possible to predict the next evolutionary step for a population using detailed knowledge of the mutation and fitness landscape for the population in a given environment. To know what variation is available to a

population, we need to know the single step mutational landscape (the probability of each mutation to occur) and the effect of these mutations on the fitness of the organism (fitness landscape). Limited information on the mutational landscape might already inform evolutionary predictions (e.g., knowledge of epistasis for key mutations can predict evolutionary pathways (Salverda *et al.* 2011)).

Obtaining the single step mutational landscape with the accompanying fitness effects is a hard problem given the sheer scale of it. Even if we only focus on a single mutational step, and assume that the environment is stable, the number of possible mutations is very high (~>10^9 in eukaryotes) which makes it hard to determine the fitness effect for each mutation. Several approaches have been used to try and tackle parts of this problem, by focussing on small genome viruses (Tisthammer *et al.* 2020), mutational scanning of one gene (Lee *et al.* 2018), or on a small metabolic pathway (Kemble *et al.* 2020). Mutation accumulation experiments can also be used to understand the mutational landscape although they are unlikely to capture all mutations (Sane *et al.* 2018). For any prediction to be relevant, we need to take into account changing environments and multiple mutations and their interactions (epistasis), although there is some indication that environmental change does not change the fitness landscape completely (Vos *et al.* 2018).

Nevertheless, progress has been made through a combination of two approaches: the statistical approach where measured fitness effects are correlated with specific mutations (top-down) (Wang *et al.* 2018) and a mechanistic approach to predict by reconstructing the genotype-phenotype-fitness map (bottom-up) (de Vos *et al.* 2015). For instance, all the epistatic interactions between mutations affecting the expression of two genes in a linear metabolic pathway were resolved with a mechanistic perspective taking into account the flux in the pathway, the toxicity of the intermediate metabolite and the protein expression cost (Kemble *et al.* 2020). At a more integrated level, the well-established polarity network in budding yeast was predictive of mutation effects (Daalman & Laan 2020). Both approaches have their own advantages and disadvantages, and in most cases both approaches will need to be combined to make the best prediction.

3. (Microbial) metabolic and growth models

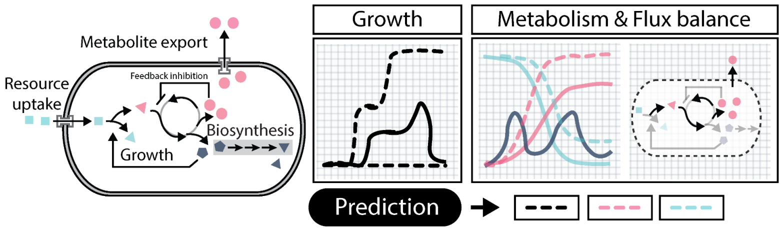

In many environments, selection on microorganisms is differential population growth of alternative genotypes, and therefore metabolism and growth are directly linked to fitness. This means we can use metabolic and growth models to predict evolution. The most well-known predictions are growth rates and metabolic adaptations in new environments (e.g. in *E. coli* (Schuetz *et al.* 2007) and *Lactobacillus plantarum* (Teusink *et al.* 2009)). These predictions use genome-scale metabolic models (Gu *et al.* 2019), which predict metabolic enzymes from genome information, constrain uptake fluxes with experimental data

and optimise for growth rate with flux balance analysis (Orth *et al.* 2010). Genome-scale metabolic models are also used for biotechnological applications, for example to predict how knock-outs of specific enzymes affect growth and product formation (see the Box in the main text)

Microbial growth and metabolism models come in different levels of detail. A simple phenomenological model can predict fitness increase (Wiser *et al.* 2013) and a more detailed model with intracellular 'macro'-reactions can be used to predict antibiotic resistance evolution (Pinheiro *et al.* 2021), or overflow metabolism (Molenaar *et al.* 2009, Wortel *et al.* 2016). Much more detailed models include genome-scale metabolic models (GEMs, current status in (Gu *et al.* 2019)), metabolism and expression models (ME models, (O'Brien *et al.* 2013)), resource balance analysis models (RBA models, (Goelzer *et al.* 2015)) and the most extensive whole cell models (Karr *et al.* 2012). Flux Balance analysis can be used to predict fluxes that lead to optimal growth, constrained by measured maximal fluxes, in GEMs, and therefore as a prediction of evolution . To improve predictions, proteome and kinetic constraints can be added (Sánchez *et al.* 2017, Chen & Nielsen 2021), or detailed descriptions of protein-metabolite dynamics can be incorporated at the expense of decreasing the model size (Wortel *et al.* 2018).

4. Population-genetic models

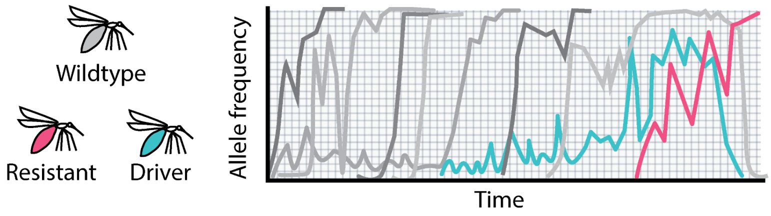

Population-genetic models are models that keep track of the genetic status (often at one or a few loci) of an entire population (Hartl 2020). Population genetic models can include mutation, fitness, reproduction, recombination and other parameters, with both deterministic and stochastic forces. These models are used widely to predict allele frequencies, e.g. incidence of deleterious recessive alleles in human populations (e.g. due to mutation/selection balance), or of conditionally beneficial alleles (e.g. sickle cell anaemia alleles in areas with Malaria parasites). Additionally, they can be used as a null model, to detect signatures of selection within population genomics, or deviations from panmixia (e.g. population differentiation).

Population-genetic models predict the evolutionary dynamics of allelic variants of genes. They use population-genetic theory, to describe the frequencies of alternative alleles (at one or multiple loci) over time. New alleles can be created from the wildtype allele by mutation, or may get introduced through immigration. Alleles at multiple sites can be recombined in following generations.

However, population genetic theory only works well for predicting changes in allele frequencies or population sizes if it is known which mutations have an effect on fitness and what these effects are (though see the extensive literature on adaptive walks (Gillespie

1983, Orr 1998)). Inferring the distribution of fitness effects of new mutations is difficult because of the sheer number of possible mutations and because this distribution may differ between genetic backgrounds and between different environments. Moreover, the mutations of interest, which are the beneficial ones, are only a small fraction of the possible mutations. Using population genetic theory to predict evolution relies either on the availability of large data sets capturing the genetic variation in natural populations (e.g. (Eyre-Walker & Keightley 2007, Tataru *et al.* 2017)) or on extensive measurements of fitness effects of selected mutations in the laboratory (e.g., (Fowler & Fields 2014, Cote-Hammarlof *et al.* 2021)).

Both Mendelian and non-Mendelian inheritance can be incorporated into the models. The models follow a population of individuals, to assess the separate or combined effects of mutation, selection, drift, non-random mating and migration, to calculate expectations for which allele(s) will spread, stabilise in frequency or disappear. In the absence of any of the evolutionary processes, allele frequencies are expected to remain largely constant and genotype frequencies can be accurately predicted, as described by the Hardy-Weinberg principle. If a beneficial allele is present in the population (from standing genetic variation, immigration or due to new mutations), it is expected to become more common in the population due to natural selection, and the models can predict the rate of change and the eventual equilibrium frequency to which the allele frequencies in the population will evolve. Ultimately, these models specify the particulars of the various deterministic and stochastic processes that operate concurrently, and how these processes contribute to the resulting evolutionary dynamics.

One interesting case study of population genetic models for predicting evolution is the evolutionary dynamics of gene drive systems (Unckless *et al.* 2017). This is explained in more detail in Box 1. In this model the frequencies of three alleles are followed over time. The alleles are (1) the **wildtype allele** (which is susceptible to the gene drive system), (2) the **driver allele** (which can convert the wildtype allele into a driver allele and may come with a fitness cost, in particular when the gene drive is used to control a population) and (3) a **resistant allele**, which can be created from the wildtype allele by mutation, and cannot be converted to the driver allele. An additional layer of complexity that was included in the model by Unckless *et al.* is that the CRISPR/Cas9 gene drive system itself is mutagenic, because the cuts it makes in chromosomes are often repaired by the non-homologous end-joining pathway which leads to mutations. The population-genetic model showed that if we want gene drive systems to be successful, we need to either reduce the rate at which resistance alleles are created, or we need to drastically increase the cost of resistance. Both of these methods have now successfully been used in experimental populations (Noble *et al.* 2017, Champer *et al.* 2020).

5. Quantitative genetics and the breeder's equation

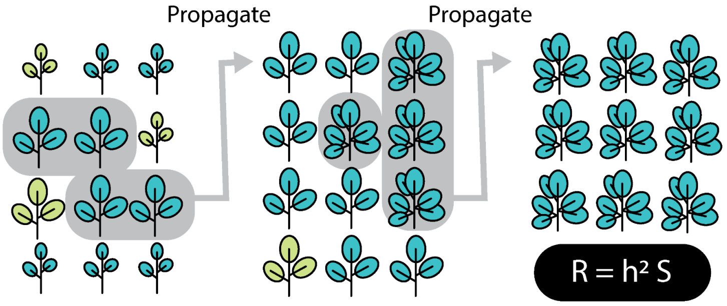

The field of quantitative genetics was developed in close association with animal and plant breeders. In situations where the focus is on altering one or a few phenotypes, over short timescales and in relatively controlled environments, it has achieved great predictive success. For example, plant-breeding programs that are designed to improve crop yield focus on creating variable populations of hybrids and selecting for plants with the highest yield (under normal or stressed conditions) (Cooper *et al.* 2014, Gaffney *et al.* 2015, Masuka *et al.* 2017). Thanks to quantitative genetics, it is well understood that faster improvements will be attained when there is either more genetic variation to start with, or stronger selection. Specific predictions are achieved through the application of general statistical tools, often under simple assumptions of polygenicity and additivity (the infinitesimal model), to large sample sizes (Barton *et al.* 2017).

While application of the 'Breeder's equation' to predict the response of single traits, from their heritability and a defined selection differential, has yielded encouraging results (Walsh & Lynch 2018), p. 607), multivariate selection often fails, even in the controlled environment of the laboratory (Milocco & Salazar-Ciudad 2020, Rouzic *et al.* 2020) reviewed in (Roff 2007), though see (Beldade *et al.* 2002, Bolstad *et al.* 2015). In the wild, our aspirations must currently be limited to a number of exceptional, closely-studied systems such as the red deer on the Isle of Rum in Scotland (Bonnet *et al.* 2019), Soay sheep on St Kilda island (Clutton-Brock *et al.* 1991), and bird populations such as great and blue tits (Charmantier *et al.* 2008, 2014, 2016).

6. Epidemiological models (SIR models)

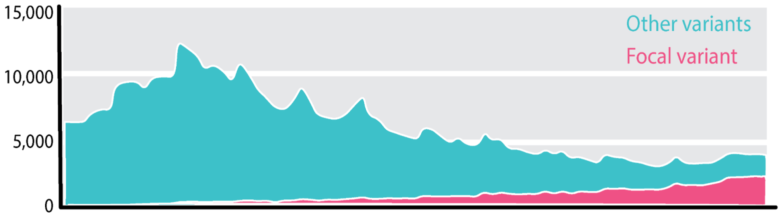

Classical SIR models are used to model the spread of an infectious agent in a population with susceptible, infected and recovered individuals. When we include the possibility of the pathogen to evolve (e.g., changing virulence or infection probabilities), we can predict the spread of an evolving infectious agent (Gordo *et al.* 2009). This type of model has been applied to modelling of influenza evolution (Boni *et al.* 2006).

In the most simple case, evolution in SIR models can be modelled by including two or more versions of the pathogen with different parameters such as the replication number or transmission probability. Such models are used for the SARS-CoV-2 pandemic to predict case numbers in the near future and the spread of new variants. In early 2021, two main virus variants affected the predictions of cases in the near future in Europe: the “wildtype” strain and the alpha variant. In many locations, a simultaneous decline of the wildtype virus and increase of the alpha variant resulted in an initial reduction of total cases, followed by an increase (see figure). When enough sequencing data was available and R_0 was known for both variants, this U-shaped pattern of cases over time could accurately be predicted. When predictions are made further into the future, they need to account for the possibility that new, currently unknown, variants will emerge (Cobey 2020, Day *et al.* 2020, Kissler *et al.* 2020).

7. Species distributions across space and environmental conditions

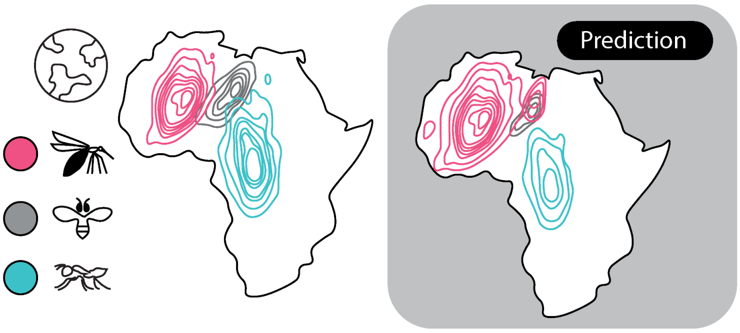

Forecasting biodiversity responses to climate change are generally done through species distribution models, which include niche, envelope and bioclimatic models (Waldvogel *et al.* 2020). These models have been used in so-called rewilding, i.e. conservation efforts that include ecological restoration and reintroduction of predators and keystone species. However, species distribution models usually do not include intraspecific variation, adaptive plasticity and evolutionary potential (Jay *et al.* 2012, Fitzpatrick & Edelsparre 2018), and therefore greatly underestimate species range dynamics. Alternative models that include genomic data and evolutionary responses have been developed to predict the (potential) range expansion of *Aedes aegypti* mosquitoes transmitting dengue virus (Kearney *et al.* 2009), to predict coral adaptation to future ocean warming (Bay *et al.* 2017) and to predict future population declines of yellow warblers (*Setophaga petechia*) and to guide effective mitigation efforts for these birds (Bay *et al.* 2018). With models that include genomic data from a species, genomic variation can be related to environmental variables (Fitzpatrick & Keller 2015). This information can then be used to predict how vulnerable populations are to environmental change, such as climate change.

8. Multi-scale evolutionary modelling

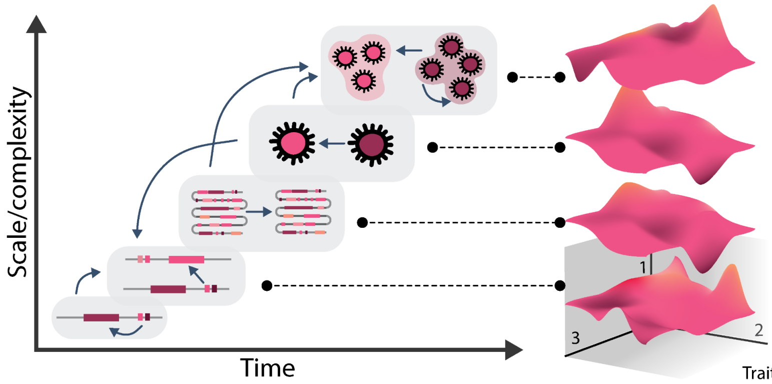

Predictions to test fundamental assumptions of evolving systems often require predictions over very long timescales. These predictions are complicated by the fact that the local mutational landscape changes with the accumulation of many new mutations, and the genotype-phenotype map changes as evolutionary innovations occur. Studying long-term evolution therefore requires models in which the genotype-phenotype map can evolve. An evolvable genotype-phenotype map can be achieved by including more than one level of organisation in a model and allowing for the evolution of traits at multiple spatiotemporal scales, leading to multi-scale evolutionary models.

Examples of multi-scale models are: the coupling of large gene regulatory networks to tissue-level patterning, which have been used for hindcasting the order of evolutionary innovations in bilateral animals (Vroomans *et al.* 2016), and estimating the likelihood that mutations increase morphological complexity (Hagolani *et al.* 2021); models of genome evolution (Cuypers & Hogeweg 2012), which predict that genomically complex ancestors primarily adapted through gene loss during major radiation events (Deutekom et al. 2019); and agent based models with rudimentary genomes, which predict emergent selective forces that can drive major evolutionary transitions (Colizzi *et al.* 2020). Multi-scale models can also generate predictions for clinically relevant evolutionary problems, such as HIV evolution (Doekes *et al.* 2017) or tumour progression (Szabó & Merks 2017).

A different scale on which selection can act, is at the level of the community (Doulcier *et al.* 2020, Chang *et al.* 2021). Predicting evolution at the level of communities is a newly emerging field with possible applications in medicine, biotechnology and agriculture. A promising application is for microbial communities that degrade hazardous compounds.

Multi-scale models are also used to study the predictability of evolution. This may either be done directly, e.g., to determine under what conditions evolution is predictable (Meijer *et al.* 2020), and show that although evolutionary paths and detailed characteristics can be hard to predict, in some cases population level traits are predictable (van Dijk *et al.* 2019). More indirectly, they can predict properties of the evolutionary landscape that affect predictability (see Section 2 of main text). E.g., common predictions of multi-scale models are that ruggedness and smoothness coexist in high dimensional fitness landscapes (e.g. arising from many-genes interactions), and the distribution of fitness effects is biased to high neutrality and high lethality, but with few intermediately deleterious mutants (Hogeweg 2012).

9. Machine learning

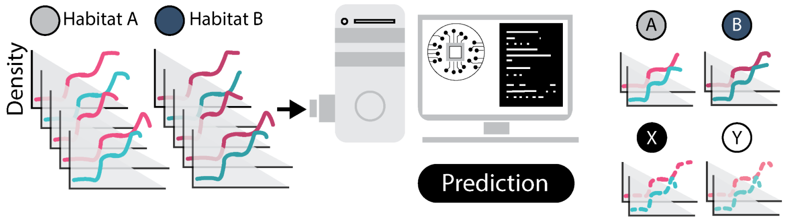

In cases where large amounts of data on repeated evolutionary trajectories in the past are available, machine learning approaches are likely to become increasingly important for predicting evolution. However, depending on the particular machine learning approach taken, these methods may or may not increase our understanding of the underlying evolutionary forces. An important limitation for using machine learning to make predictions is that it requires data from a very similar situation because the predictions can usually not be extended to novel situations beyond the data set used for training, in contrast to mechanistic models. One interesting example of machine learning for evolutionary predictions is a study on experimental evolution using the integration of numerous evolve and resequence experiments with *E. coli*. The researchers developed a predictor of the genes that will be modified in the course of adaptation, depending on the *E. coli* strain and the selection pressure (Wang *et al.* 2018). The model could predict around one third of the mutational targets in a new but similar evolve and resequence experiment.

Machine learning methods have also been used to predict the somatic evolution of cancer (Caravagna *et al.* 2018, Gerhauser *et al.* 2018) and success of influenza virus variants (Hayati *et al.* 2020). A particularly promising future direction is the ability for machine learning methods to combine increasingly complex cancer genomic data with data on transcriptome, epigenome and advanced imaging to guide precision medicine (Gerstung *et al.* 2020).

References

Barton, N. H., A. M. Etheridge, and A. Véber. 2017. The infinitesimal model: Definition, derivation, and implications. Theor. Popul. Biol. 118: 50–73.

Bay, R. A., R. J. Harrigan, V. L. Underwood, H. L. Gibbs, T. B. Smith, and K. Ruegg. 2018. Genomic signals of selection predict climate-driven population declines in a migratory bird. Science 359: 83–86.

Bay, R. A., N. H. Rose, C. A. Logan, and S. R. Palumbi. 2017. Genomic models predict successful coral adaptation if future ocean warming rates are reduced. Sci. Adv. 3: e1701413.

Beldade, P., K. Koops, and P. M. Brakefield. 2002. Developmental constraints versus flexibility in morphological evolution. Nature 416: 844–847.

Bolstad, G. H., J. A. Cassara, E. Márquez, T. F. Hansen, K. van der Linde, D. Houle, and C. Pélabon. 2015. Complex constraints on allometry revealed by artificial selection on the wing of Drosophila melanogaster. Proc. Natl. Acad. Sci. 112: 13284–13289.

Boni, M. F., J. R. Gog, V. Andreasen, and M. W. Feldman. 2006. Epidemic dynamics and antigenic evolution in a single season of influenza A. Proc. R. Soc. B Biol. Sci. 273: 1307–1316.

Bonnet, T., M. B. Morrissey, A. Morris, S. Morris, T. H. Clutton-Brock, J. M. Pemberton, and L. E. B. Kruuk. 2019. The role of selection and evolution in changing parturition date in a red deer population. PLOS Biol. 17: e3000493.

Bull, J. J., and I. J. Molineux. 2008. Predicting evolution from genomics: experimental evolution of bacteriophage T7. Heredity 100: 453–463.

Burke, M. K., and M. R. Rose. 2009. Experimental evolution with Drosophila. Am. J. Physiol.-Regul. Integr. Comp. Physiol. 296: R1847–R1854.

Caravagna, G., Y. Giarratano, D. Ramazzotti, I. Tomlinson, T. A. Graham, G. Sanguinetti, and A. Sottoriva. 2018. Detecting repeated cancer evolution from multi-region tumor sequencing data. Nat. Methods 15: 707–714.

Champer, J., E. Yang, E. Lee, J. Liu, A. G. Clark, and P. W. Messer. 2020. A CRISPR homing gene drive targeting a haplolethal gene removes resistance alleles and successfully spreads through a cage population. Proc. Natl. Acad. Sci. 117: 24377–24383.

Chang, C.-Y., J. C. C. Vila, M. Bender, R. Li, M. C. Mankowski, M. Bassette, J. Borden, S. Golfier, P. G. L. Sanchez, R. Waymack, X. Zhu, J. Diaz-Colunga, S. Estrela, M. Rebolleda-Gomez, and A. Sanchez. 2021. Engineering complex communities by directed evolution. Nat. Ecol. Evol. 1–13.

Charmantier, A., C. Doutrelant, G. Dubuc-Messier, A. Fargevieille, and M. Szulkin. 2016. Mediterranean blue tits as a case study of local adaptation. Evol. Appl. 9: 135–152.

Charmantier, A., D. Garant, and L. E. B. Kruuk. 2014. Quantitative Genetics in the Wild. OUP Oxford.

Charmantier, A., R. H. McCleery, L. R. Cole, C. Perrins, L. E. B. Kruuk, and B. C. Sheldon. 2008. Adaptive Phenotypic Plasticity in Response to Climate Change in a Wild Bird Population. Science 320: 800–803.

Chen, Y., and J. Nielsen. 2021. Mathematical modeling of proteome constraints within metabolism. Curr. Opin. Syst. Biol. 25: 50–56.

Clutton-Brock, T. H., O. F. Price, S. D. Albon, and P. A. Jewell. 1991. Persistent Instability and Population Regulation in Soay Sheep. J. Anim. Ecol. 60: 593–608.

Cobey, S. 2020. Modeling infectious disease dynamics. Science 368: 713–714.

Colizzi, E. S., R. M. Vroomans, and R. M. Merks. 2020. Evolution of multicellularity by collective integration of spatial information R. E. Goldstein and A. M. Walczak (Eds.). eLife 9: e56349.

Cooper, M., C. D. Messina, D. Podlich, L. R. Totir, A. Baumgarten, N. J. Hausmann, D. Wright, and G. Graham. 2014. Predicting the future of plant breeding: complementing empirical evaluation with genetic prediction. Crop Pasture Sci. 65: 311.

Cote-Hammarlof, P. A., I. Fragata, J. Flynn, D. Mavor, K. B. Zeldovich, C. Bank, and D. N. A. Bolon. 2021. The Adaptive Potential of the Middle Domain of Yeast Hsp90. Mol. Biol. Evol. 38: 368–379.

Cuypers, T. D., and P. Hogeweg. 2012. Virtual Genomes in Flux: An Interplay of Neutrality and Adaptability Explains Genome Expansion and Streamlining. Genome Biol. Evol. 4: 212–229.

Daalman, W. K.-G., and L. Laan. 2020. Predicting an epistasis-rich genotype-phenotype map with a coarse-grained bottom-up model of budding yeast polarity. bioRxiv 2020.11.09.374363.

Day, T., S. Gandon, S. Lion, and S. P. Otto. 2020. On the evolutionary epidemiology of SARS-CoV-2. Curr. Biol. 30: R849–R857.

van Dijk, B., J. Meijer, T. D. Cuypers, and P. Hogeweg. 2019. Trusting the hand that feeds: microbes evolve to anticipate a serial transfer protocol as individuals or collectives. BMC Evol. Biol. 19: 201.

Doekes, H. M., C. Fraser, and K. A. Lythgoe. 2017. Effect of the Latent Reservoir on the Evolution of HIV at the Within- and Between-Host Levels. PLOS Comput. Biol. 13: e1005228.

Doulcier, G., A. Lambert, S. De Monte, and P. B. Rainey. 2020. Eco-evolutionary dynamics of

nested Darwinian populations and the emergence of community-level heredity W. Shou, P. J. Wittkopp, W. Shou, S. Mitri, and A. Sanchez (Eds.). eLife 9: e53433.

EDWARDS, C. E. 2015. Looking to the future of conservation genetics: The case for using quantitative genetic experiments to estimate the ability of rare plants to withstand climate change. Am. J. Bot. 102: 1011–1013.

EYRE-WALKER, A., and P. D. KEIGHTLEY. 2007. The distribution of fitness effects of new mutations. Nat. Rev. Genet. 8: 610–618.

FITZPATRICK, M. C., and S. R. KELLER. 2015. Ecological genomics meets community-level modelling of biodiversity: mapping the genomic landscape of current and future environmental adaptation. Ecol. Lett. 18: 1–16.

FITZPATRICK, M. J., and A. H. EDELSPARRE. 2018. The genomics of climate change. Science 359: 29–30.

FOWLER, D. M., and S. FIELDS. 2014. Deep mutational scanning: a new style of protein science. Nat. Methods 11: 801–807.

GAFFNEY, J., J. SCHUSSLER, C. LÖFFLER, W. CAI, S. PASZKIEWICZ, C. MESSINA, J. GROETEKE, J. KEASCHALL, and M. COOPER. 2015. Industry-Scale Evaluation of Maize Hybrids Selected for Increased Yield in Drought-Stress Conditions of the US Corn Belt. Crop Sci. 55: 1608–1618.

GERHAUSER, C. ET AL. 2018. Molecular Evolution of Early-Onset Prostate Cancer Identifies Molecular Risk Markers and Clinical Trajectories. Cancer Cell 34: 996-1011.e8.

GERSTUNG, M. ET AL. 2020. The evolutionary history of 2,658 cancers. Nature 578: 122–128.

GILLESPIE, J. H. 1983. A simple stochastic gene substitution model. Theor. Popul. Biol. 23: 202–215.

GOELZER, A., J. MUNTEL, V. CHUBUKOV, M. JULES, E. PRESTEL, R. NÖLKER, M. MARIADASSOU, S. AYMERICH, M. HECKER, P. NOIROT, D. BECHER, and V. FROMION. 2015. Quantitative prediction of genome-wide resource allocation in bacteria. Metab. Eng. 32: 232–243.

GORDO, I., M. G. M. GOMES, D. G. REIS, and P. R. A. CAMPOS. 2009. Genetic Diversity in the SIR Model of Pathogen Evolution. PLOS ONE 4: e4876.

GU, C., G. B. KIM, W. J. KIM, H. U. KIM, and S. Y. LEE. 2019. Current status and applications of genome-scale metabolic models. Genome Biol. 20: 121.

HAGOLANI, P. F., R. ZIMM, R. VROOMANS, and I. SALAZAR-CIUDAD. 2021. On the evolution and development of morphological complexity: A view from gene regulatory networks. PLOS Comput. Biol. 17: e1008570.

HARTL, D. L. 2020. A Primer of Population Genetics and Genomics 4th ed. Oxford University Press, Oxford.

HAYATI, M., P. BILLER, and C. COLIJN. 2020. Predicting the short-term success of human influenza virus variants with machine learning. Proc. R. Soc. B Biol. Sci. 287: 20200319.

HOGEWEG, P. 2012. Toward a Theory of Multilevel Evolution: Long-Term Information Integration Shapes the Mutational Landscape and Enhances Evolvability. *In* O. S. Soyer (Ed.) Evolutionary Systems Biology. Advances in Experimental Medicine and Biology. pp. 195–224, Springer, New York, NY.

JAY, F., S. MANEL, N. ALVAREZ, E. Y. DURAND, W. THUILLER, R. HOLDEREGGER, P. TABERLET, and O. FRANÇOIS. 2012. Forecasting changes in population genetic structure of alpine plants in response to global warming. Mol. Ecol. 21: 2354–2368.

JONES, F. C. ET AL. 2012. The genomic basis of adaptive evolution in threespine sticklebacks. Nature 484: 55–61.

KARR, J. R., J. C. SANGHVI, D. N. MACKLIN, M. V. GUTSCHOW, J. M. JACOBS, B. BOLIVAL, N. ASSAD-GARCIA, J. I. GLASS, and M. W. COVERT. 2012. A Whole-Cell Computational Model Predicts Phenotype from Genotype. Cell 150: 389–401.

KAWECKI, T. J., R. E. LENSKI, D. EBERT, B. HOLLIS, I. OLIVIERI, and M. C. WHITLOCK. 2012. Experimental evolution. Trends Ecol. Evol. 27: 547–560.

KEARNEY, M., W. P. PORTER, C. WILLIAMS, S. RITCHIE, and A. A. HOFFMANN. 2009. Integrating biophysical models and evolutionary theory to predict climatic impacts on species'

ranges: the dengue mosquito Aedes aegypti in Australia. Funct. Ecol. 23: 528–538.

Kemble, H., C. Eisenhauer, A. Couce, A. Chapron, M. Magnan, G. Gautier, H. L. Nagard, P. Nghe, and O. Tenaillon. 2020. Flux, toxicity, and expression costs generate complex genetic interactions in a metabolic pathway. Sci. Adv. 6: eabb2236.

Kissler, S. M., C. Tedijanto, E. Goldstein, Y. H. Grad, and M. Lipsitch. 2020. Projecting the transmission dynamics of SARS-CoV-2 through the postpandemic period. Science 368: 860–868.

Lee, J. M., J. Huddleston, M. B. Doud, K. A. Hooper, N. C. Wu, T. Bedford, and J. D. Bloom. 2018. Deep mutational scanning of hemagglutinin helps predict evolutionary fates of human H3N2 influenza variants. Proc. Natl. Acad. Sci. 115: E8276–E8285.

Lind, P. A. 2019. Repeatability and Predictability in Experimental Evolution. *In* P. Pontarotti (Ed.) Evolution, Origin of Life, Concepts and Methods. pp. 57–83, Springer International Publishing, Cham. Available at: https://doi.org/10.1007/978-3-030-30363-1_4 [Accessed November 3, 2020].

Masuka, B., G. N. Atlin, M. Olsen, C. Magorokosho, M. Labuschagne, J. Crossa, M. Bänziger, K. V. Pixley, B. S. Vivek, A. von Biljon, J. Macrobert, G. Alvarado, B. M. Prasanna, D. Makumbi, A. Tarekegne, B. Das, M. Zaman-Allah, and J. E. Cairns. 2017. Gains in Maize Genetic Improvement in Eastern and Southern Africa: I. CIMMYT Hybrid Breeding Pipeline. Crop Sci. 57: 168–179.

Meijer, J., B. van Dijk, and P. Hogeweg. 2020. Contingent evolution of alternative metabolic network topologies determines whether cross-feeding evolves. Commun. Biol. 3: 1–13.

Milocco, L., and I. Salazar-Ciudad. 2020. Is evolution predictable? Quantitative genetics under complex genotype-phenotype maps. Evolution 74: 230–244.

Molenaar, D., R. van Berlo, D. de Ridder, and B. Teusink. 2009. Shifts in growth strategies reflect tradeoffs in cellular economics. Mol. Syst. Biol. 5: 323.

Nadell, C. D., K. Drescher, and K. R. Foster. 2016. Spatial structure, cooperation and competition in biofilms. Nat. Rev. Microbiol. 14: 589–600.

Noble, C., J. Olejarz, K. M. Esvelt, G. M. Church, and M. A. Nowak. 2017. Evolutionary dynamics of CRISPR gene drives. Sci. Adv. 3: e1601964.

O'Brien, E. J., J. A. Lerman, R. L. Chang, D. R. Hyduke, and B. Ø. Palsson. 2013. Genome-scale models of metabolism and gene expression extend and refine growth phenotype prediction. Mol. Syst. Biol. 9: 693.

Orr, H. A. 1998. THE POPULATION GENETICS OF ADAPTATION: THE DISTRIBUTION OF FACTORS FIXED DURING ADAPTIVE EVOLUTION. Evol. Int. J. Org. Evol. 52: 935–949.

Orth, J. D., I. Thiele, and B. Ø. Palsson. 2010. What is flux balance analysis? Nat. Biotechnol. 28: 245–248.

Pinheiro, F., O. Warsi, D. I. Andersson, and M. Lässig. 2021. Metabolic fitness landscapes predict the evolution of antibiotic resistance. Nat. Ecol. Evol. 1–11.

Remigi, P., C. Masson-Boivin, and E. P. C. Rocha. 2019. Experimental Evolution as a Tool to Investigate Natural Processes and Molecular Functions. Trends Microbiol. 27: 623–634.

Roff, D. A. 2007. A Centennial Celebration for Quantitative Genetics. Evolution 61: 1017–1032.

Rouzic, A. L., C. Renneville, A. Millot, S. Agostini, D. Carmignac, and É. Édeline. 2020. Unidirectional response to bidirectional selection on body size II. Quantitative genetics. Ecol. Evol. 10: 11453–11466.

Sackman, A. M., L. W. McGee, A. J. Morrison, J. Pierce, J. Anisman, H. Hamilton, S. Sanderbeck, C. Newman, and D. R. Rokyta. 2017. Mutation-Driven Parallel Evolution during Viral Adaptation. Mol. Biol. Evol. 34: 3243–3253.

Salverda, M. L. M., E. Dellus, F. A. Gorter, A. J. M. Debets, J. van der Oost, R. F. Hoekstra, D. S. Tawfik, and J. A. G. M. de Visser. 2011. Initial Mutations Direct Alternative Pathways of

Protein Evolution. PLOS Genet. 7: e1001321.

Sánchez, B. J., C. Zhang, A. Nilsson, P. Lahtvee, E. J. Kerkhoven, and J. Nielsen. 2017. Improving the phenotype predictions of a yeast genome-scale metabolic model by incorporating enzymatic constraints. Mol. Syst. Biol. 13.

Sane, M., J. J. Miranda, and D. Agashe. 2018. Antagonistic pleiotropy for carbon use is rare in new mutations. Evolution 72: 2202–2213.

Schenk, M. F., M. P. Zwart, S. Hwang, P. Ruelens, E. Severing, J. Krug, and J. A. G. M. de Visser. 2022. Population size mediates the contribution of high-rate and large-benefit mutations to parallel evolution. Nat. Ecol. Evol. 6: 439–447.

Schuetz, R., L. Kuepfer, and U. Sauer. 2007. Systematic evaluation of objective functions for predicting intracellular fluxes in Escherichia coli. Mol. Syst. Biol. 3: 119.

Szabó, A., and R. M. H. Merks. 2017. Blood vessel tortuosity selects against evolution of aggressive tumor cells in confined tissue environments: A modeling approach. PLOS Comput. Biol. 13: e1005635.

Tataru, P., M. Mollion, S. Glémin, and T. Bataillon. 2017. Inference of Distribution of Fitness Effects and Proportion of Adaptive Substitutions from Polymorphism Data. Genetics 207: 1103–1119.

Teotónio, H., S. Estes, P. C. Phillips, and C. F. Baer. 2017. Experimental Evolution with Caenorhabditis Nematodes. Genetics 206: 691–716.

Teusink, B., A. Wiersma, L. Jacobs, R. A. Notebaart, and E. J. Smid. 2009. Understanding the Adaptive Growth Strategy of Lactobacillus plantarum by In Silico Optimisation. PLOS Comput. Biol. 5: e1000410.

Tisthammer, K. H., W. Dong, J. B. Joy, and P. S. Pennings. 2020. Assessing in vivo mutation frequencies and creating a high-resolution genome-wide map of fitness costs of Hepatitis C virus. bioRxiv 2020.10.01.323253.

Unckless, R. L., A. G. Clark, and P. W. Messer. 2017. Evolution of Resistance Against CRISPR/Cas9 Gene Drive. Genetics 205: 827–841.

de Vos, M. G. J., A. Dawid, V. Sunderlikova, and S. J. Tans. 2015. Breaking evolutionary constraint with a tradeoff ratchet. Proc. Natl. Acad. Sci. 112: 14906–14911.

Vos, M. G. J. de, S. E. Schoustra, and J. A. G. M. de Visser. 2018. Ecology dictates evolution? About the importance of genetic and ecological constraints in adaptation. EPL Europhys. Lett. 122: 58002.

Vroomans, R. M. A., P. Hogeweg, and K. H. W. J. ten Tusscher. 2016. In silico evo-devo: reconstructing stages in the evolution of animal segmentation. EvoDevo 7: 14.

Waldvogel, A.-M., B. Feldmeyer, G. Rolshausen, M. Exposito-Alonso, C. Rellstab, R. Kofler, T. Mock, K. Schmid, I. Schmitt, T. Bataillon, O. Savolainen, A. Bergland, T. Flatt, F. Guillaume, and M. Pfenninger. 2020. Evolutionary genomics can improve prediction of species' responses to climate change. Evol. Lett. 4: 4–18.

Walsh, B., and M. Lynch. 2018. Evolution and Selection of Quantitative Traits. Oxford University Press.

Wang, X., V. Zorraquino, M. Kim, A. Tsoukalas, and I. Tagkopoulos. 2018. Predicting the evolution of Escherichia coli by a data-driven approach. Nat. Commun. 9: 3562.

Wiser, M. J., N. Ribeck, and R. E. Lenski. 2013. Long-Term Dynamics of Adaptation in Asexual Populations. Science 342: 1364–1367.

Wortel, M. T., E. Bosdriesz, B. Teusink, and F. J. Bruggeman. 2016. Evolutionary pressures on microbial metabolic strategies in the chemostat. Sci. Rep. 6: 29503.

Wortel, M. T., E. Noor, M. Ferris, F. J. Bruggeman, and W. Liebermeister. 2018. Metabolic enzyme cost explains variable trade-offs between microbial growth rate and yield. PLOS Comput. Biol. 14: e1006010.

Zandbergen, L. E., T. Halverson, J. K. Brons, A. J. Wolfe, and M. G. J. de Vos. 2021. The Good and the Bad: Ecological Interaction Measurements Between the Urinary Microbiota and Uropathogens. Front. Microbiol. 12.
